# Studying the Effects of Oral Contraceptives on Coagulation Using a Mathematical Modeling Approach

**DOI:** 10.1007/978-3-031-58516-6_4

**Published:** 2024-03-28

**Authors:** Amy Kent, Karin Leiderman, Anna C. Nelson, Suzanne S. Sindi, Melissa M. Stadt, Lingyun (Ivy) Xiong, Ying Zhang

**Affiliations:** Mathematical Institute, University of Oxford, Oxfordshire, UK; Mathematics Department, Computational Medicine Program, University of North Carolina at Chapel Hill, Chapel Hill, NC, USA; Department of Mathematics, Duke University, Durham, NC, USA; Department of Applied Mathematics, University of California Merced, Merced, CA, USA; Department of Applied Mathematics, University of Waterloo, Waterloo, ON, Canada; Department of Qualitative and Computational Biology, University of Southern California, Los Angeles, CA, USA; Department of Mathematics, Brandeis University, Waltham, MA, USA

## 1 Introduction

Exogenous hormones are used by hundreds of millions of people worldwide for contraceptives and hormonal replacement therapy. Hormonal contraceptives contain either exclusively progestin—a synthetic progesterone—or a combination of progestin and estrogen in the form of ethinyl estradiol. Combined oral contraceptives (OCs) are classified by the type of progestin and the level of estrogen dose used in the formulation, where the action of progestin prevents ovulation by suppressing luteinizing hormone and estrogen prevents breakthrough bleeding [1]. Progestins used in OCs are grouped by “generations” that correspond to when they first appeared in the formulation. For example, second-generation progestins were used in the 1970s and include levonorgestrel and norgestrel, and third-generation progestins introduced in the 1990s include gestodene, norgestimate, and desogestrel [1, 2].

The use of combined, or combination, oral contraceptives (OCs) and hormone replacement therapies is known to increase the risk of both arterial and venous thrombosis (pathological blood clot formation) [3–8]. While the estrogen component of combination OCs is known to be prothrombotic [4, 5], the progestin formulation has also been shown to affect clotting propensity [9, 10]. Indeed, studies suggest that for a fixed estrogenic dosage, patients on a third-generation OC containing desogestrel and gestoden have a higher risk for venous thrombosis than patients on second-generation OCs that use progestins, such as levonorgestrel and norethisterone [11]. However, the mode of delivery of the OC does not affect the risk for thrombosis, as transdermal and transvaginal forms of contraception also show an increased risk of thrombosis [12, 13]. Exogenous hormones from combined OCs can modulate components of the procoagulant, anticoagulant, and fibrinolytic components of blood coagulation [14–17]. One example is the changes in plasma levels of clotting factors when using OCs [17]. These alterations may elicit a prothrombotic state that is dependent on dose of estrogen and hormonal dose combination [18, 19]. Individuals with deficiencies in endogenous anticoagulant proteins are also more susceptible to thrombosis when taking combination OCs [10, 20, 21]. How various modulations to the clotting system mechanistically contribute to an increased thrombosis risk is not fully understood.

Clotting factors that are modulated while on OCs are components of the blood coagulation network, which is responsible for the generation of the important clotting enzyme thrombin. Blood coagulation involves inhibitors, both positive and negative feedback loops, and must exhibit a robust clotting response given a wide variety of factor levels. Due to these complexities, mathematical modeling can be used to better understand how modulations like exogenous hormones can affect this process. In this study, we are particularly interested in modeling the clotting factors involved in blood coagulation. Our mathematical model uses factor concentration as an input and outputs thrombin concentration over time. For a review of the variety of mathematical models used to describe various components of the blood clotting process, see [22].

One measure that is known to correlate with thrombosis risk is the resistance of the clotting system to the inhibitory effects of activated protein C (APC) [9, 23, 24]. APC is an anticoagulant protein generated during coagulation that serves as a brake on the clotting system to prevent over clotting and spreading of clotting to areas beyond an injury. A clotting system that is more resistant to the effects of APC could therefore be more prothrombotic. The use of OCs is associated with an increased APC resistance [16, 25–27], with patients on third-generation (desogestrel-based) OCs having a more pronounced APC resistance than patients on second-generation (levonorgestrel-based) OCs [16]. Taken together, the use of OCs is thus associated with an increased risk of thrombosis, and the APC resistance (or sensitivity) is one metric to predict this risk.

There are different ways to test for APC resistance, but the most common ways are by comparing an activated partial thromboplastin time (APTT) or an endogenous thrombin potential (ETP) with and without APC [27]. The complete details of these assays are beyond the scope of this chapter, but essentially they test the timing and strength of a clotting response. de Visser et al. [27] performed both kinds of APC resistance tests on hundreds of patients, some of whom were on OCs and some of whom were not. Their study suggested that, in general, clotting factor VIII (FVIII) and clotting factor II (FII, also known as prothrombin), to a lesser extent, are primary determinants of the outcomes in APTT-based tests and that the clotting inhibitor tissue factor pathway inhibitor (TFPI) and protein S are primary determinants in ETP-based tests. Additionally, they suggested that the use of OCs only moderately affected the APTT-based test but strongly affected the ETP-based test and that in the latter case the effects may not be due to clotting factor levels alone. The comparisons were performed on patients on and off OCs, but they were not the same individuals. Furthermore, the correlations were computed using single clotting factors; thus, they would not be able to capture simultaneous contributions from multiple factors.

Midderdorp et al. [17] studied the effects of OCs on clotting factor levels in an elegant cross-over study that reported levels of six clotting factors in 28 patients off OCs and on levogestrel (lev) and desogestrel. This study provided information about how factor levels are *changed* by the OCs, which enables further study regarding the link between OCs, factor levels, and thrombosis risk. However, what was not reported in the paper was the individual patient changes, rather just mean and standard deviation of the study cohorts.

In the current study, we used the data from the Middeldorp et al. [17] study, together with a mathematical model of coagulation, to investigate how factor level changes from OCs affect production and timing of the coagulation enzyme thrombin in addition to an APC sensitivity metric. Because the individual factor level changes were not reported in the Middeldorp et al. [17] study, we used the reported means and standard deviations to generate a large virtual patient population (VPP). We then simulated the effects of lev by adjusting the factor levels by the mean effect of lev reported in [17]. We analyzed the concentration and timing of thrombin generation among the entire VPP after the use of lev and reported the characteristics of patients that had large and small changes in outputs. We computed APC sensitivity ratios and showed that the use of lev, by way of factor level changes alone, increased the systems’ sensitivity to APC. Our results suggest that factor changes induced by lev are enough to explain both a change in APC sensitivity and an increased prothrombotic profile.

## 2 Methods

### 2.1 Brief Review of Mathematical Model of Flow-Mediated Coagulation

Here we give a brief review of a mathematical model of flow-mediated coagulation [28–32] on which we build for the current study. A more detailed description of the model can be found in the supplementary information at the end of this chapter along with the full model equations in Eqs. (4)–(122), parameters in Tables 2, 3, 4, 5, 6, 7, and 8, and a schematic of the flow-mediated coagulation model in Fig. 12. Further details about this model and its sensitivity to parameters can be found elsewhere [33]. Briefly, the model simulates blood coagulation and platelet deposition under flow. Blood coagulation is a network of biochemical reactions that culminate in the production of the enzyme thrombin. Platelet deposition and aggregation is a biophysical process that initially stops leakage of blood from a vessel. Thrombin is generated on the platelet surfaces and then cleaves the soluble protein fibrinogen into fibrin that turns into a gel and stabilizes the platelets. Here, we are assuming a very small injury completely contained in a blood vessel.

**Fig. 1:**
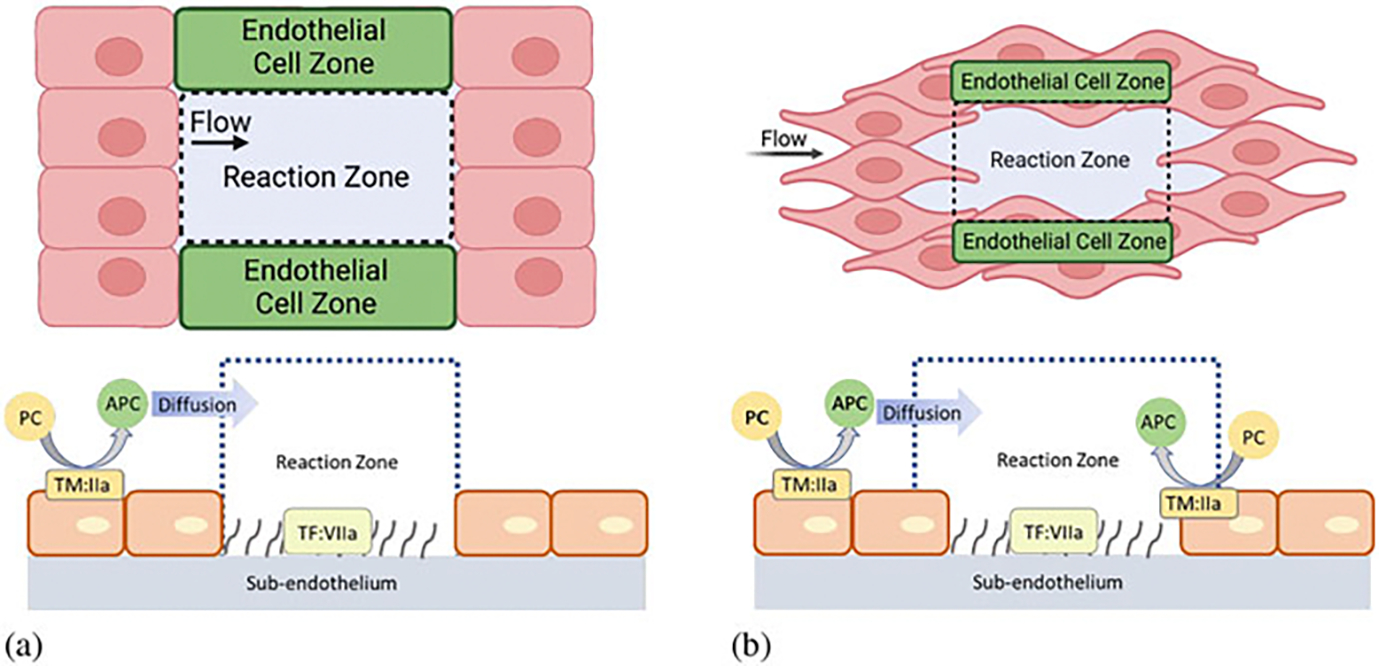
Model schematics: (**a**) the top and side view of the reaction zone (RZ) and endothelial zone (EZ) in our previous models and (**b**) the updated zones in our current study. Our previous model had distinct RZ and EZ zones that relied on thrombin from the RZ to diffuse to the EZ to make APC, and then the APC had to diffuse back into the RZ to have an inhibitory effect. In the new model, due to protruding ECs into the RZ, thrombin and APC can be generated together in the RZ

The model simulates the coagulation reactions and platelet deposition at a small injury patch with exposed tissue factor, all occurring in flowing blood (Fig. 12). The reactions occur in two main compartments: the reaction zone (RZ) and the endothelial zone (EZ). Represented schematically in Fig. 1a, the RZ compartment models the region above an injury site, and the EZ compartment models the surrounding region, introduced to account for the effects of flow-mediated transport. Each compartment is assumed to be well-mixed; thus, the time evolution of the concentration of all species is modeled using ordinary differential equations. Different variables are introduced to account for the platelet-bound, membrane-bound, and free concentrations of the relevant enzymes and zymogens (enzyme precursors) within each compartment. The height of the RZ is given by the length scale where diffusive and advective transport are comparable, and the width is taken to be the characteristic size of an intravascular injury, i.e., 10 microns [28]. The EZ height is taken to be the same as the RZ, and the width is dependent on the flow shear rate and protein diffusion coefficients [29]. The coagulation reactions occur in the RZ, where tissue factor (TF) in the subendothelium (SE) is exposed, as depicted in Fig. 12b. The clotting factors, denoted by Roman numerals, and platelets are transported into and out of the RZ. This is represented in the model by a simplified combination of flow and diffusion in the form of a mass transfer coefficient, which characterizes the flow-mediated transport between the two compartments. Clotting factor concentrations in the RZ change due to their involvement in reactions and by transport in and out of the zone. Platelet concentrations are treated similarly. But as platelets build up in the RZ, they are assumed to cover and hinder the enzymatic activity on the subendothelium, and they alter the height and volume of the RZ. The EZ is located adjacent to the RZ, in the direction perpendicular to the flow with height equal to that of the RZ. In the EZ, thrombin that has diffused from the RZ can bind to thrombomodulin (TM) and then protein C in the EZ and activate protein C into APC. This APC either diffuses back into the RZ or is carried away by the flow. APC in the RZ can bind to and inactivate FVa and FVIIIa, which may slow thrombin generation. The inhibitory effect depends on how much FVa and FVIIIa are already in a complex (FVa binds to FXa, and FVIIIa binds FIXa on platelet surfaces) because once bound, they are protected from APC.

### 2.2 Model Extension: Thrombomodulin and APC Generation in the Reaction Zone

TM is located on endothelial cell (EC) membranes and is generally not embedded in the SE, hence the development of separate compartments in our previous models. Here, we have assumed that portions of ECs protrude into the RZ, thereby providing some mixing of the two zones and their contents. Under this assumption, thrombin in the RZ could readily access and bind to the TM in the RZ, creating active complexes to generate APC *within* the RZ. Compared to our previous models, where APC entered the RZ compartment only by diffusion [29, 31], this assumption should lead to more APC that is available to inactivate FVa and FVIIIa in the RZ. We also assume that platelets cannot cover and inhibit the activity on these protruding EC surfaces, i.e., APC generation occurs even after platelets have covered the subendothelium. Details of the EZ and RZ in models, old and new, are shown schematically in Fig. 1. In the previous model, APC diffusion into the RZ was modeled as $k_{flow}^{c}\left(c_{out}-c\right)$, where c is the concentration in the RZ, $c_{out}$ is the upstream concentration, and $k_{flow}^{c}$ is the rate constant for flow-mediated transport, related to the diffusion coefficient, lumen velocity, and injury size as given in [29].

In this chapter, we allow for protrusion of EC debris into the injury zone. EC protrusion into the RZ compartment allows for direct generation of APC in the RZ *via* TM, in addition to the flow-mediated transport considered previously. To account for the protrusion of EC into the injury zone within the model, we introduce TM and its associated complexes into the RZ compartment. Three new species were added to the model: TM in the RZ ${TM}_{rz}$, TM in the RZ that is in complex with thrombin ${TM}_{rz}:E_{2}$, and the complex of TM, thrombin, and protein $C\;{TM}_{rz}:E_{2}:PC$. The reactions involved are

(1) $${TM}_{rz}+E_{2}\underset{k^{-}}{\overset{k^{+}}{⇌}}{TM}_{rz}:E_{2},$$

(2) $${TM}_{rz}:E_{2}+PC\underset{k_{pc}^{-}}{\overset{k_{pc}^{+}}{⇌}}{TM}_{rz}:E_{2}:PC\overset{k_{pc}^{cat}\;}{\to }{TM}_{rz}:E_{2}+APC,$$

where Reaction (1) is the binding of thrombin to TM in the RZ and Reaction (2) is the binding of thrombin-bound TM to protein C and the subsequent enzymatic cleavage into activated protein C (APC). The kinetic rates in these reactions were taken from a study using the previous mathematical model [29]. The model extension impacts on the model equations are highlighted as bold underlined terms in Eqs. (23), (40), (121), and (122).

### 2.3 Virtual Patient Population Generation

Based on the data presented in [17], we generated a large virtual patient population. We first generated the smooth kernel density estimates for the population distribution of clotting factors II, V, VII, VIII, and X for patients not on OCs from reported data from [17]. Kernel density functions allowed us to estimate an underlying probability distribution from a sample (see [34] for details). We used the MATLAB function ksdensity to create our kernel density estimates independently for each factor level.

**Fig. 2:**
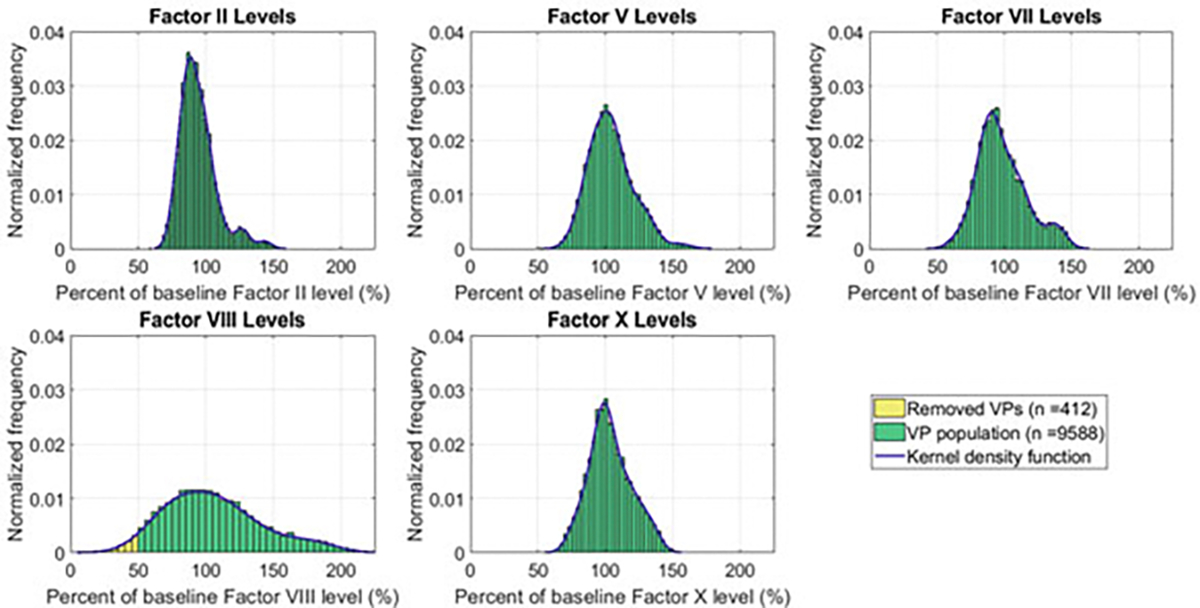
Factor levels for virtual patients (VPs) were sampled from kernel density estimates computed from data in [17]. Distributions of coagulation factor levels for the virtual patient population before taking OCs. The blue curves indicate the smooth kernel density estimates. Virtual patients with factor VIII level less than 50% were removed (n=412) and are highlighted in yellow. For factor levels while on levonorgestrel, factor levels for each virtual patient were changed by the fixed amount given in Table 1

We then created 10,000 virtual patients by randomly and independently sampling factor levels from our kernel density estimates. See Fig. 2 for factor level distributions and kernel density estimates. To determine the factor levels for each virtual patient under lev treatment, we added the mean change in each individual coagulation factor level as reported in [17] (see Table 1). Because virtual patients should represent normal, healthy individuals, we then removed any virtual patients that did not exhibit factor levels within normal physiological range. Specifically, we removed the 412 virtual patient samples that had factor VIII levels below 50% (i.e., out of normal physiological range).

### 2.4 Model Workflow

Each virtual patient had a set of unique factor levels, sampled from the kernel density estimates independently. These factor levels were then used as input (initial conditions) to our mathematical model, which consists of a system of ordinary differential equations that track how each model variable changes in time, under flow. See the supplementary information for more model details, equations, and parameters. The equations of the model were solved numerically to predict concentrations of each species through time. We mainly analyzed thrombin concentrations for this study, but all species concentrations are available for further mechanistic studies.

## 3 Results

### 3.1 Thrombomodulin in the Reaction Zone

**Fig. 3:**
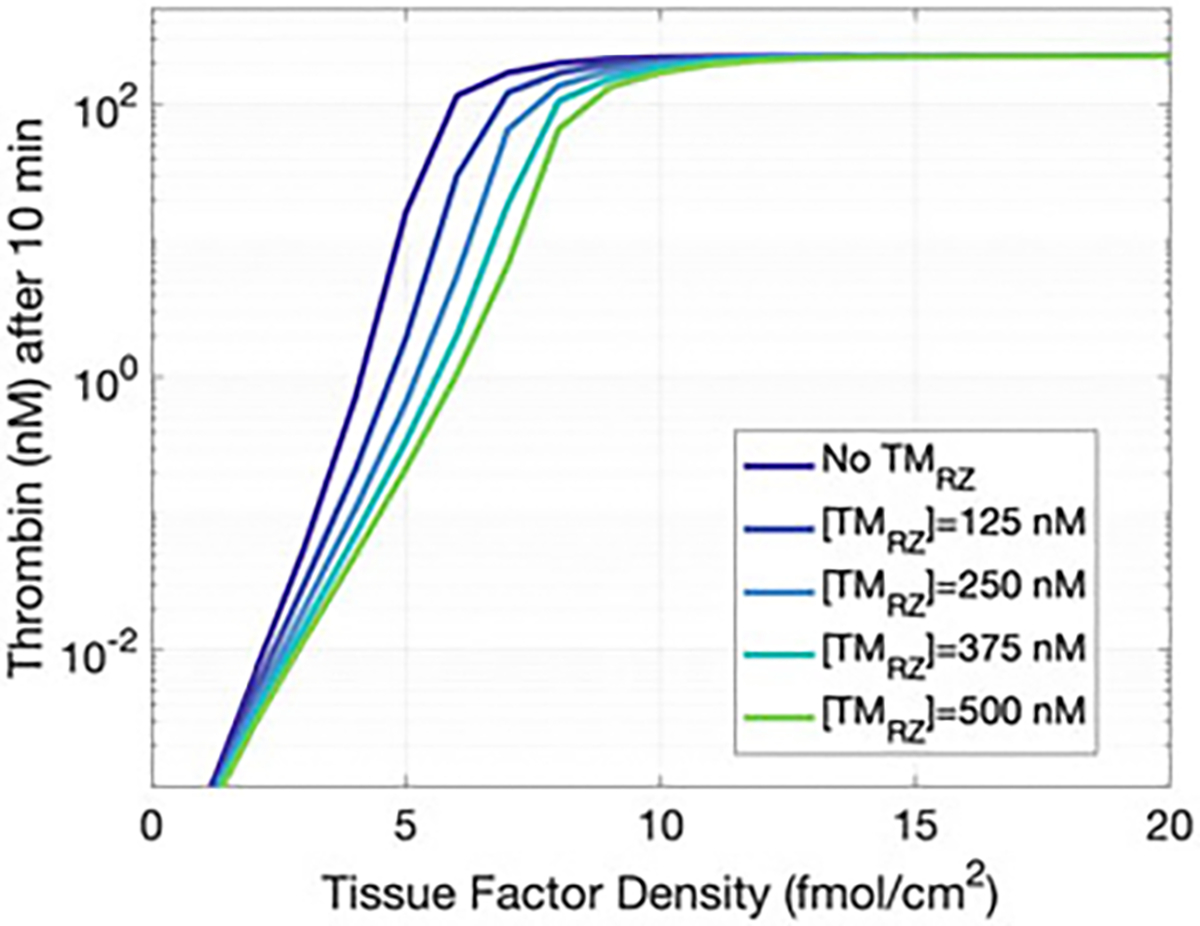
Thrombin concentration after 10 min over a range of tissue factor densities that subsequently increase activated protein C (APC) in the reaction zone (RZ). To achieve the same level of thrombin response, more tissue factor is needed as thrombomodulin (TM) is added

The addition of thrombomodulin (TM) into the RZ is described in Sect. 2.2. Briefly, TM was added to the RZ to enable APC generation by thrombin within the RZ. This feature was added to the model to enhance the sensitivity of the system to APC. Here we study how it alters the clotting dynamics. Figure 3 shows the thrombin concentration after 10 min of activity as a function of the tissue factor density. Tissue factor is the protein embedded in the subendothelium that stimulates the initiation of coagulation and thus thrombin generation. Any single curve in Fig. 3 shows the known threshold dependence of thrombin on tissue factor; the system should have a strong response only when necessary, as clotting is unwarranted without injury. Threshold plots are shown for concentrations of TM in the RZ varying between zero and the concentration assumed in the EZ (500 nM [29]). As the concentration of TM in the RZ increases, an increased tissue factor density is required to attain the same thrombin concentration as we expect, since the APC generation in the RZ has increased. APC inactivates FVa and FVIIIa, which inhibits the formation of two key complexes in the coagulation pathway (Fig. 12): FXa:FVa (prothrombinase), which activates prothrombin to thrombin, and FIXa:FVIIIa (tenase), which activates FX to FXa.

**Fig. 4:**
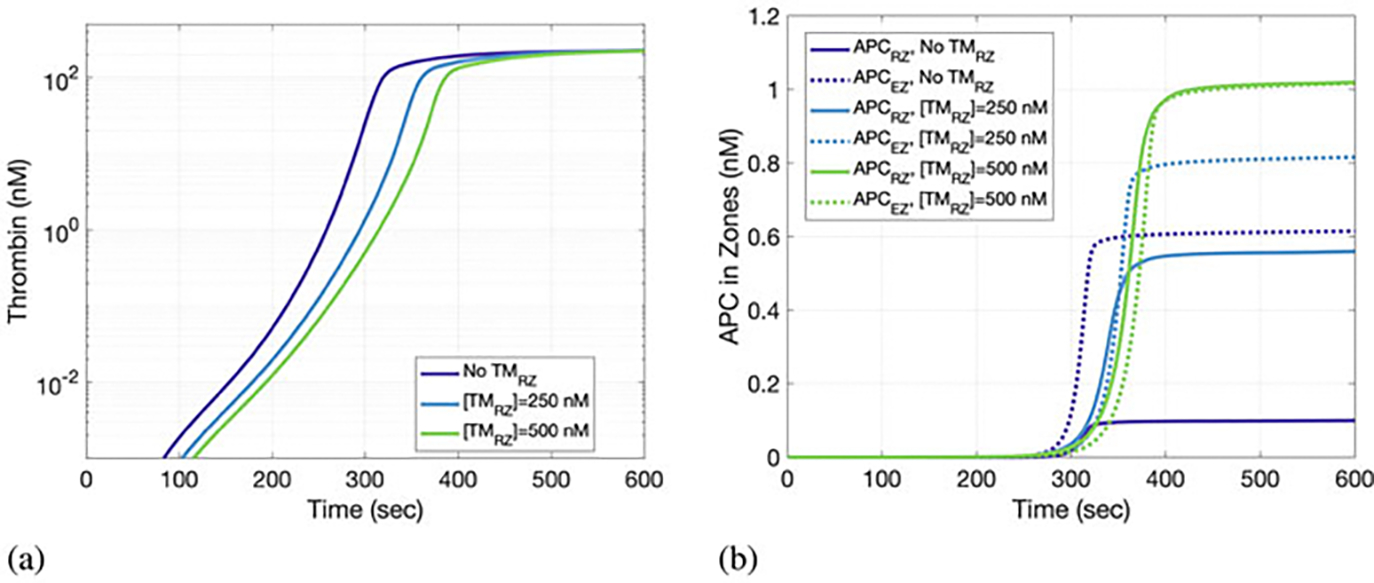
The effects of thrombomodulin (TM) in the reaction zone (RZ) on (**a**) thrombin and (**b**) activated protein C (APC) concentrations in the RZ and endothelial zone (EZ) over 10 min of clotting activity. TM in the RZ leads to increased thrombin generation and increased APC in the RZ

**Fig. 5:**
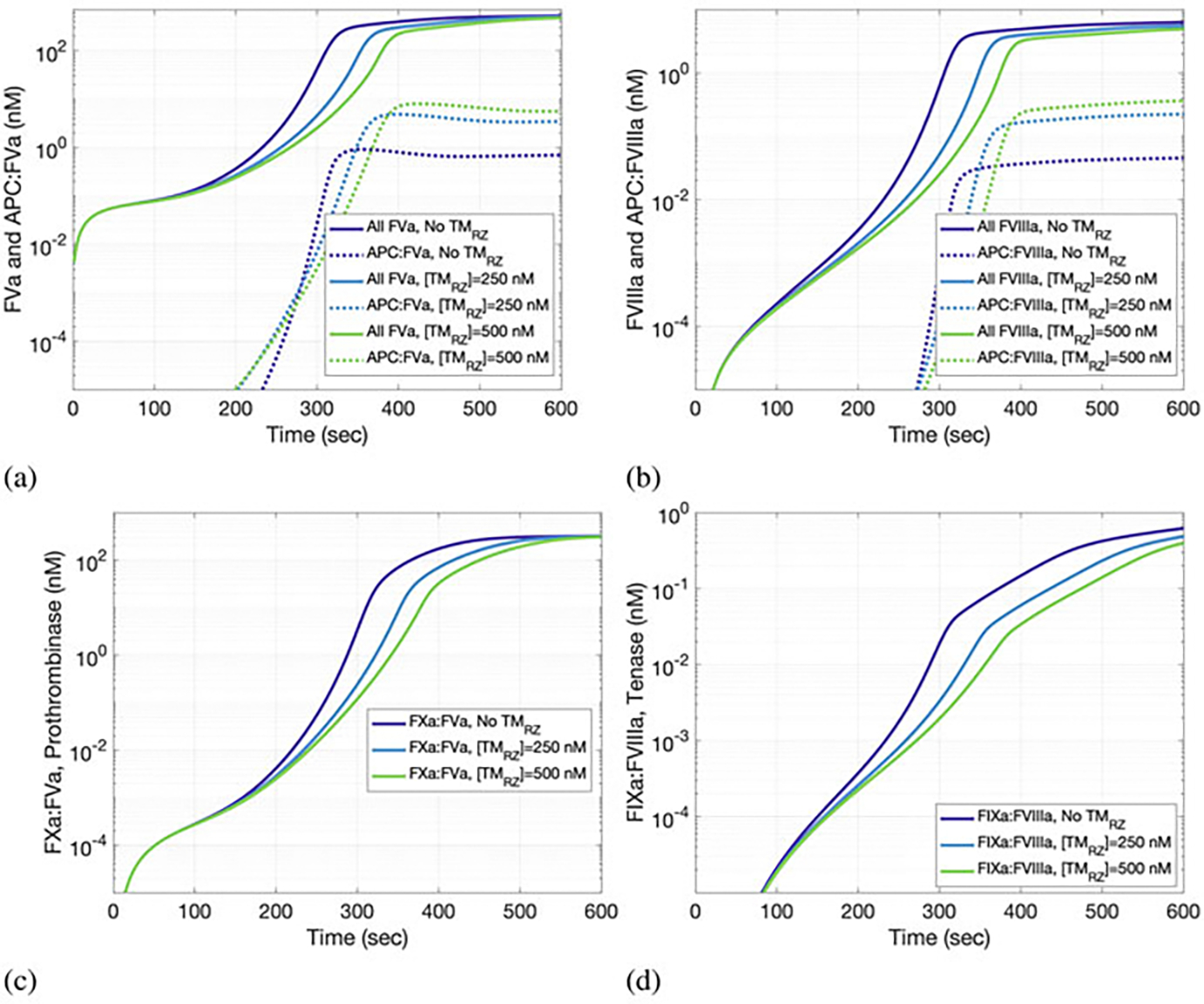
The effects of activated protein C (APC) generation in the reaction zone (RZ) on (**a**) FVa, (**b**) FVIIIa, (**c**) FXa:FVa, and (**d**) FIXa:FVIIIa over 10 min of clotting activity. APC generation in the RZ leads to inactivation of FVa and FVIIIa and a reduction of prothrombinase and tenase

The effects of increased APC generation on clotting dynamics can be explored by considering the time evolution of different factors (Figs. 4 and 5). In Fig. 4, the thrombin lag time (i.e., the time when 1 nM thrombin is generated) increases with increased concentration of TM in the RZ. This is likely due to increased TM in the RZ and the inhibitory effects of the associated increases in APC. Increased APC generation arising from the introduction of TM in the RZ is confirmed in Fig. 4. When no TM is present in the RZ, APC is transported into the RZ from the EZ by diffusion alone, as shown in the model schematic (Fig. 1). Introducing TM into the RZ, thereby allowing APC generation directly within the RZ, increases the amount of total APC generated (Fig. 4).

APC causes a reduction in thrombin generation *via* its inhibitory effects. The specific effect by inactivating factors Va and VIIIa is illustrated in Fig. 5, where the time evolution of total and APC-bound factors Va and VIIIa is shown. Increasing the concentration of TM in the RZ increases the proportion of activated Va and VIIIa.

In summary, the model extension of TM in the RZ led to APC in the RZ that inactivated FVa and FVIIIa, thereby limiting the formation of VIIIa:IXa and Va:Xa complexes, which directly and negatively affected the generation of thrombin. Having established that this extension results in a sensible clotting response, in the next section we use the model to simulate the clotting response of a cohort of virtual patients.

### 3.2 Predicted Effects of Levonorgestrel on Thrombin Generation

**Fig. 6:**
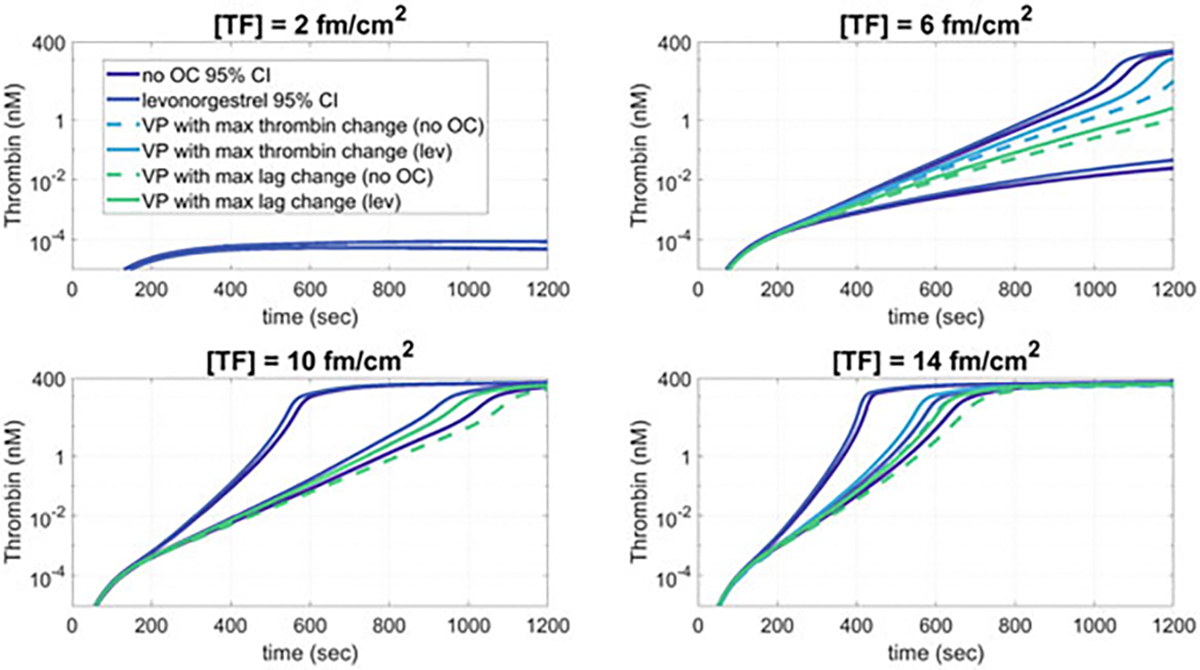
Simulated thrombin shows an upward and leftward shift on average for patients on lev, with decreased lag times and increased thrombin at 20 min. Simulated thrombin concentration time series results for virtual patients (.n=9,588) with no oral contraceptive (no OC) and levonorgestrel (lev) over 20 min for varied concentrations of tissue factor as given. The 95% confidence intervals (CIs) for the virtual patients on no OC and on lev are shown. The virtual patient individuals with the maximal change in thrombin on no OC and on lev as well as the maximal change in lag time are also plotted. Virtual patient factor level distributions are shown in Fig. 2

We performed simulations of thrombin generation over 20 min for each of the virtual patients before OC (no OC condition) and after taking lev (lev condition). The mean and 95% confidence intervals of the time series results for select tissue factor (TF) levels are shown in Fig. 6. We can see that at low TF density, i.e., [TF] = 2 fmol/cm^2^, thrombin generation is minimal, which is in line with the TF threshold behavior of thrombin. This is true for virtual patients on no OCs and on lev. For higher TF levels, thrombin generation increases with increased TF concentrations, again as predicted by the threshold behavior of thrombin on TF.

For TF levels greater than 2 fmol/cm^2^, the factor level changes due to lev have a trend that shifts the thrombin curves up and to the left, which means a higher average thrombin concentration at the end of the simulation as well as a shorter lag time (i.e., the time to reach 1 nM) as compared to the same patients on no OC.

**Fig. 7:**
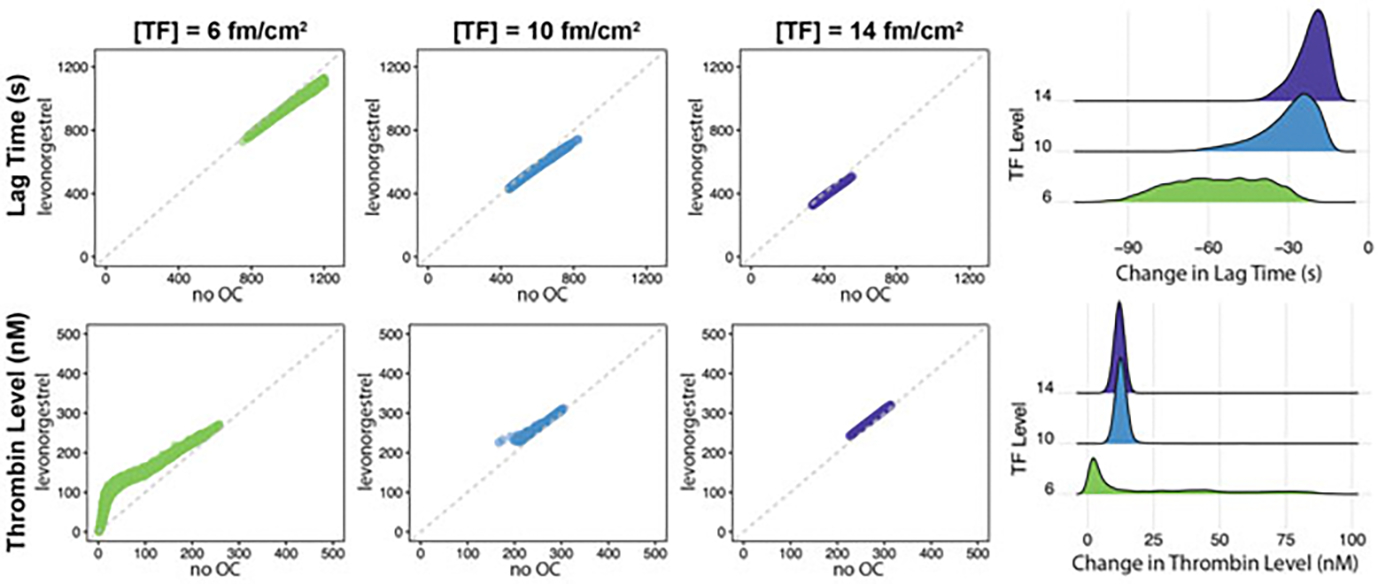
Use of levonorgestrel (lev) heightens thrombosis response. Scatter plots on the left compare lag time (top) and thrombin concentration after 20 min (bottom) before and after lev usage, for varying concentrations of tissue factor (TF) levels: 6 fmol/cm^2^ (n=4,755), 10 fmol/cm^2^ (n=9,588), and 14 fmol/cm^2^ (n=9,588). Dashed diagonal line indicates matching outcomes before and after lev usage. Density plots on the right show changes in corresponding metrics upon lev usage

Although average behavior of the population showed a trend of increased thrombin and decreased lag time, we further explored the behavior on an individual patient level. First, we collected all virtual patients whose thrombin levels reached 1 nM within 20 min, for the TF levels of 6, 10, and 14 fmol/cm^2^. Next, we examined the changes in lag time and thrombin concentration at 20 min before and after lev usage for each individual virtual patient collected. Figure 7 shows the lag time (top row) and thrombin concentration (bottom row), with these metrics for each individual on lev vs. on no OC. For the lag times, all of the data points lie below the gray dashed line, indicating that all patients had a decreased lag time on lev vs. on no OC. For the thrombin concentration, all of the data points lie above the gray dashed line, indicating that all patients had an increased thrombin concentration on lev vs. on no OC. Furthermore, the largest changes occurred at the lower TF levels. These data suggest that lev induces a heightened thrombotic response.

### 3.3 Factor Levels Inducing an Extreme Response

**Fig. 8:**
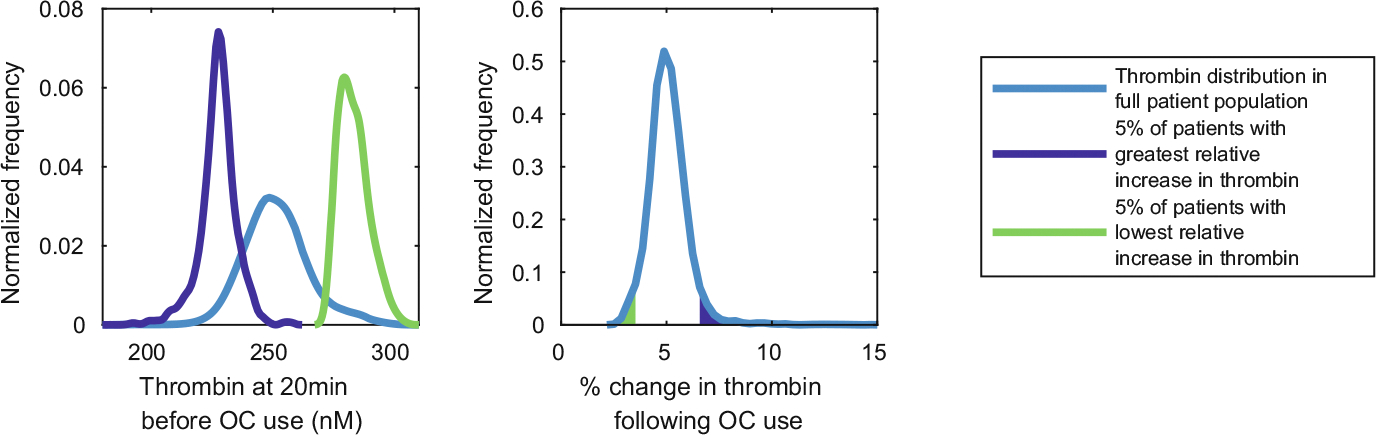
Patients with lowest thrombin generation before oral contraceptive (OC) use had the largest relative change in thrombin generation after using OCs. Left: distribution in the thrombin concentration at 20 min before OC use for the whole population and subpopulations with the greatest and smallest increases in thrombin generation following OC use. Center: distribution in the percentage change in thrombin following OC use. Tissue factor [TF] = 10 fmol/.cm^2^ for these simulations

Having established that *all* virtual patients exhibit an increase in thrombin and a decrease in lag time following the use of lev, we now turn to identifying the characteristics of patients with the most extreme changes in their thrombin metrics. For TF = 10 fmol/cm^2^, we considered the distribution of the simulated thrombin concentrations after 20 min on no OCs (the left side of Fig. 8) and the relative increase in thrombin concentration reached after 20 min when on lev compared to when on no OC (the center of Fig. 8). To do this, virtual patients were ordered by the relative increase in thrombin concentration reached after 20 min when on lev compared to when on no OC, discounting 11 patients where the simulated thrombin failed to reach 1 nM thrombin. The light blue curves represent the entire virtual patient population, and the dark blue and green curves represent subpopulations of patients that had the largest 5% and smallest 5% relative increases in thrombin after lev use. We see that the average thrombin concentration for the entire population is near 250 nM, and the mean increase in thrombin generation is 5%. We found that the largest relative changes in thrombin came from patients that had the lowest thrombin concentration prior to lev use. Similarly, the smallest relative changes in thrombin came from patients that had the highest thrombin concentration prior to lev use. This is somewhat intuitive since patients that already have strong thrombin responses prior to OC use are unlikely to have a much stronger increase after OC use. Those patients who had a smaller thrombin response prior to OC use would then likely be able to have larger relative increases in thrombin.

**Fig. 9:**
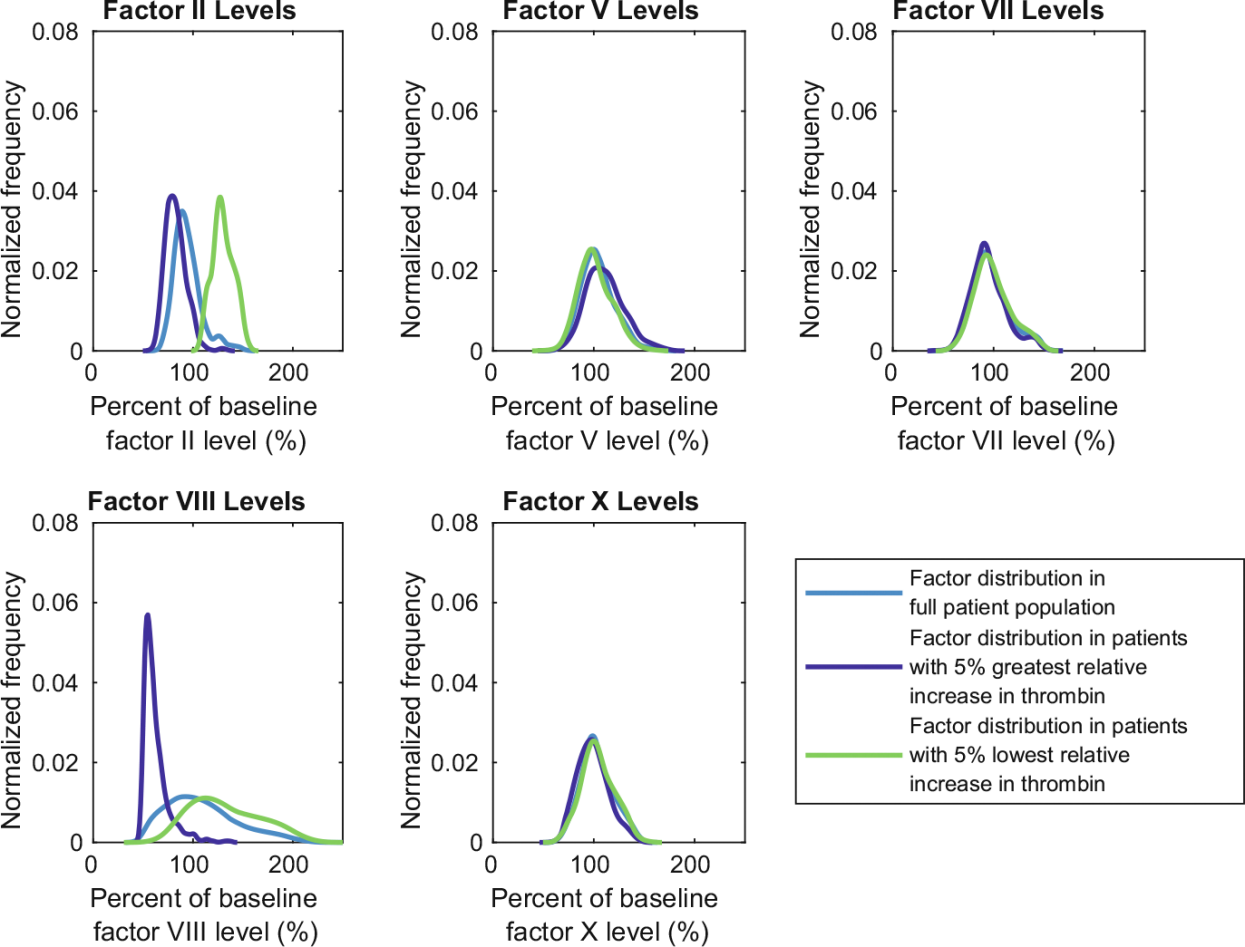
Patients with lowest levels of FVIII prior to oral contraceptive (OC) use had the largest relative increase in thrombin generation following OC use. Distribution of patient factor levels in the whole population compared with the factor distributions for the 5% of patients exhibiting the largest and smallest percentage increase in thrombin generation following OC use. All tissue factors (TFs) are reported before OC use, and [TF] = 10 fmol/.cm^2^ for the simulation of the thrombin curves that produced these percentiles

It is interesting to consider the combination of factor levels that characterize the virtual patients that experienced large relative increases in thrombin generation following OC use. We next considered the normalized distributions of factor levels in each subpopulation that exhibited the largest and smallest increases in thrombin concentration (Fig. 9). Relatively low factor VIII levels prior to OC use are observed for patients with the greatest increase in thrombin generation. Factor VIII is associated with increased thrombin generation as when activated, it binds to activated FIX on the platelet surface to form a key complex in the coagulation cascade. Given the greatest increases in thrombin generation were observed for patients with an initially low thrombin response (Fig. 8), reduced FVIII levels may be a key indicator that the virtual patient is at risk of large changes in thrombin generation following OC use.

Conversely, the patients with the smallest increase in thrombin generation following OC use were those with high prothrombin (FII) levels (Fig. 9). Prothrombin is activated by the Va:Xa complex at the platelet surface to form thrombin *in vivo*. Thus, high prothrombin levels before OC use will contribute to a larger initial thrombin concentration, which results in virtual patients with a small relative change in thrombin generation following OC use (Fig. 8).

**Fig. 10:**
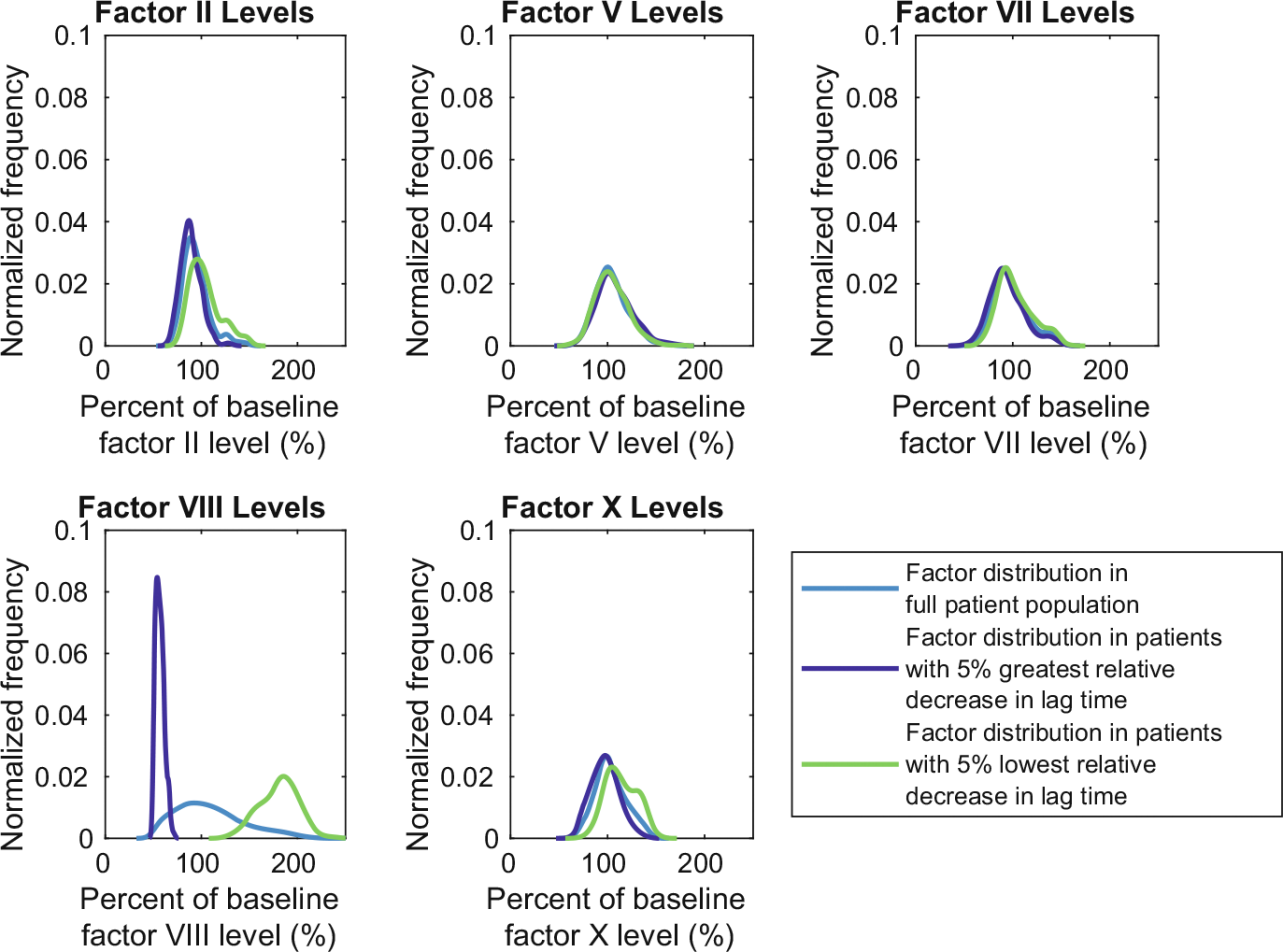
Patients with lowest levels of FVIII prior to oral contraceptives (OC) use had the largest relative decrease in lag time following OC use. Factor distributions for the 5% of patients exhibiting the greatest and smallest percentage decrease in lag time following OC use. All factors are reported before OC use with tissue factor [TF] = 10 fmol/cm^2^

Distributions in the relative change in lag time following OC use have a similar behavior; virtual patients that undergo the greatest decrease in lag time following OC are those with a lag time at baseline that is longer than average and *vice versa*. The distributions of factor levels for the 5% of virtual patients undergoing the greatest and smallest decrease in lag time are shown in Fig. 10. Low factor VIII levels again signpost the greatest change following OC use, while high factor VIII levels are associated with a small change in lag time. Elevated prothrombin levels are linked to a reduced increase in thrombin generation (Fig. 9); there is less change in thrombin since thrombin is already high with increased prothrombin. Similarly, low prothrombin levels are associated with the greatest relative increase in thrombin; this allows room for greater change in thrombin when thrombin is not as high in the first place. The effect of high prothrombin levels on decreasing changes in lag time is not as pronounced (Fig. 10), and there is no association with low prothrombin and lag time changes. Hence, while a large proportion (80%) of virtual patients are in the subpopulation undergoing the greatest 5% change for both changes in thrombin generation and lag time, factor levels that induce a large change in thrombin generation do not necessitate a large change in lag time. These results provide some insight into the factor levels that bring the greatest increase in thrombin generation and reduction in lag time. Although the TF level is relatively high in this example and there is more variance in these metrics with the lower TF (Fig. 7), the trends in factor levels and relative increases and decreases are similar (not shown). In the future work, a sensitivity analysis could be conducted to systematically identify which combinations of factors are associated with the greatest thrombotic risk.

### 3.4 APC Sensitivity Metric

To quantify the effect of APC on thrombin generation between OC and non-OC users, we developed a new APC sensitivity ratio. Our ratio is similar to the ETP-based metric [27] described in Sect. 1, because we will use an area under the curve of simulated thrombin. Ours differs from the ETP-based test in that we are not adding exogenous APC. The two cases we compare are a case with TM in the RZ (APC generation in the RZ) and no TM in the RZ (no APC generation in the RZ, so minimal effects of APC). Our metric is defined as

(3) $$APC-sr=\frac{\int_{0}^{\tau }T^{APC+OC}(t)\;dt}{\int_{0}^{\tau }T^{OC}(t)dt}/\frac{\int_{0}^{\tau }T^{APC}(t)\;dt}{\int_{0}^{\tau }T(t)\;dt},$$

where τ denotes the termination time for simulating thrombin generation, $\;T^{APC+OC}(t)$ denotes the thrombin concentration over time for the virtual patient with APC generation in the RZ and taking OC, and $\;T^{APC}(t)$ denotes the thrombin concentration over time with APC generation in the RZ but without OC. We define $\;T^{OC}(t)$ to be the thrombin concentration over time without APC generation in the RZ but with OC usage and T(t) to be the thrombin concentration over time without APC generation or OC. The ratio in the numerator of Eq. (3) gives the effect of APC when a virtual patient is on OC, whereas the ratio in the denominator of Eq. (3) represents the effect of APC when a patient is off OC. Taken together, Eq. (3) allows us to explore the inhibitory effect of APC in the presence of OC. Note that in the case where a patient does not use OC and has no APC generation in the RZ, APC-sr=1.

**Fig. 11:**
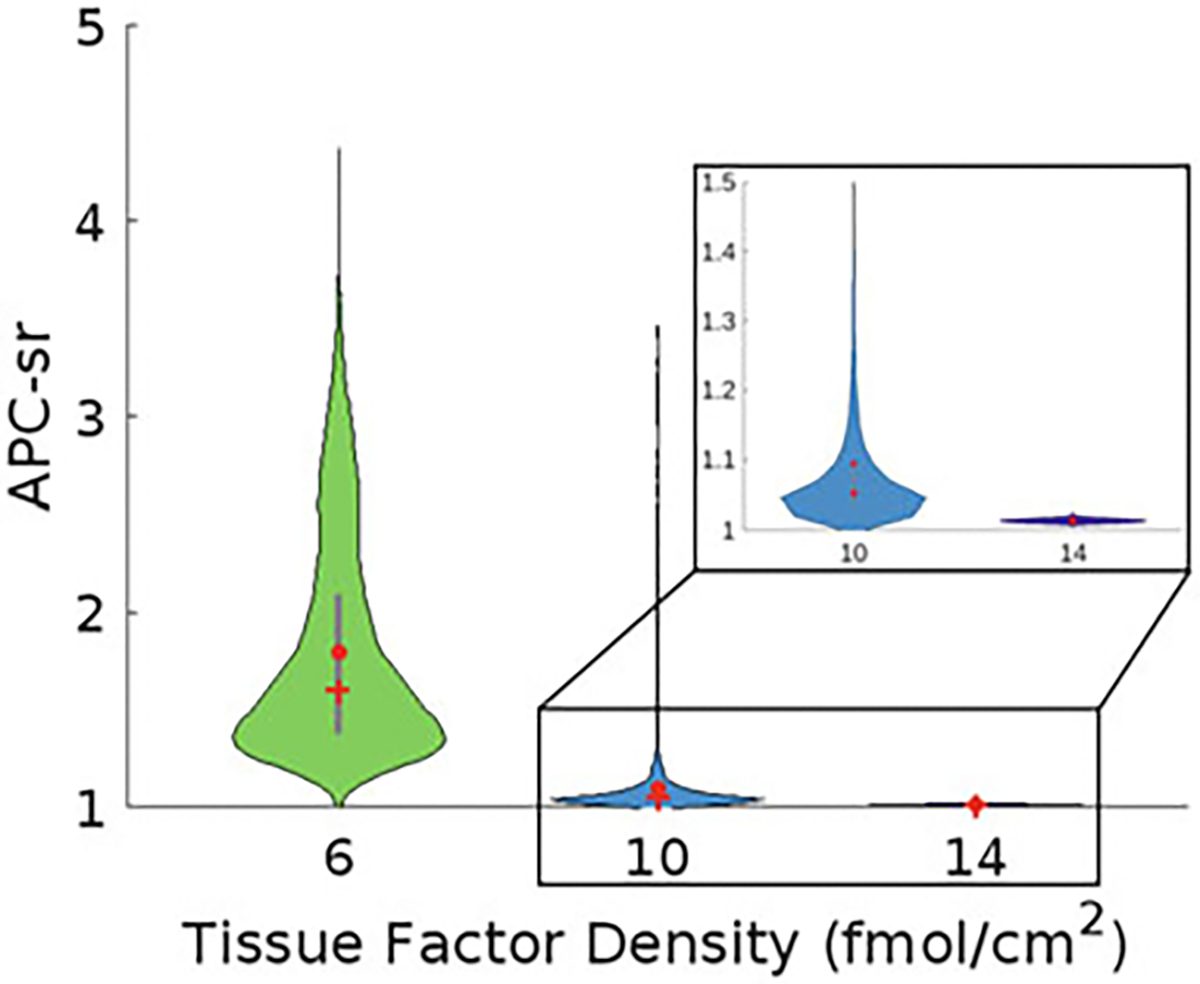
The activated protein C (APC) sensitivity ratio (APC-sr) is greater than 1 for all TF levels for patients on oral contraceptives. For TF = 6 fmol/cm^2^ (n=4,755), 10 fmol/cm^2^ (N=9,588), and 14 fmol/cm^2^ (n=9,588), the APC-sr is calculated using Eq. (3) with the sample mean represented as a solid red dot and the sample median represented as a red cross. The thick gray bar in the center represents the interquartile range. Patients with simulated thrombin curves that did not reach 1 nM thrombin by 20 min were removed from these calculations

To compute APC-sr for different TF levels, we removed virtual patients that do not reach 1 nM thrombin, so the ratio in Eq. (3) is well-defined. We set τ to be 20 min. Examining the ratios for $\left[TF\right]=6,\;10$, and 14 fmol/cm^2^ in Fig. 11, we see that the APC sensitivity ratios are increased with OC use since they are always above 1. This means that for all TF levels patients following OC use have a higher APC sensitivity (Fig. 11) than non-OC users and therefore may have an increased risk of thrombosis. In comparing the ratio as the TF level is increased, we see that APC-sr decreases on average (see red dots in Fig. 11). This shows that patients are less sensitive to APC when TF level is high.

## 4 Discussion

In this study we used a mathematical model of flow-mediated coagulation to study the effects of the OC lev on thrombin generation. To simulate the effects of the OC, we used clotting factor levels and their changes due to OC use, measured in 28 patients as part of a cross-over study [17]. Based on the clotting factor levels for the patients prior to OC use, we generated a large virtual patient population with the same mean and standard deviation as the reported data. Next, we represented the effects of the OCs on that virtual patient population by changing the clotting factor levels according to mean changes reported in the real patient data. The clotting factor levels were used as initial conditions for our mathematical model that simulates thrombin generation under flow. After analyzing the outputs of the virtual population before and after OC use, we found that the changes in clotting factor levels due to OC use always increased thrombin generation and decreased the lag time (sped up the process), with these changes being more pronounced at a low to moderate TF level. We concluded from this that the changes in factor levels alone can heighten the prothrombotic state of the clotting system in our model. Additionally, to test the system’s sensitivity to APC, we extended our previous mathematical model to include thrombomodulin, and thus APC generation within the reaction zone so that APC was not only confined to generation in the endothelial zone assumed to be distinct and adjacent to the reaction zone. In previous studies, where it was confined to the adjacent endothelial zone, APC has little to no inhibitory effect [29]. As seen in the TF threshold and thrombin plots, our model shows susceptibility to inhibition by APC. With this new model, we were then able to study the APC sensitivity that may occur with OC use. Indeed, we showed that the changes in clotting factor levels alone were enough to increase the APC sensitivity (as shown by an increased APC sensitivity ratio). Previous studies have shown only minor to moderate changes in APC sensitivity ratios due to changes in factor levels [27]. However, the assays in that study used high tissue factor levels, which possibly masked differences in relatively small factor level changes. Our model in this study focused on varied tissue factor levels to allow for more sensitivity in the system.

We have shown here that the effects of lev on our virtual patient population, in the form of clotting factor level changes alone, contribute to a prothrombotic state. However, there are some limitations of this study. We did not allow for variation in the changes with lev beyond the means reported in the data. In the future we plan to develop statistical methods to refine that assumption. Additionally, the cross-over study included data from the same patients on another OC (third generation) that we did not study here. In fact, it has been shown that the use of third-generation versus second-generation OCs is associated with an increased resistance. In the current study, we have created virtual patient populations based on the patients’ distributions of factor levels in the data, and then we changed the levels of individual patients by the same value (the mean change reported). The third-generation OC leads to a further increase in FII and FVII and a further decrease in FV. Thus, based on our results with lev, we speculate that changing the levels of the virtual patients, simply by the mean of the desogestrel data [17], should give us a similar, albeit slightly enhanced, result in terms of APC sensitivity. Investigating the effects of third-generation OCs using more sophisticated techniques to sample the data is a primary focus of our immediate future work.

## Supplementary Material

1

## Figures and Tables

**Table 1:** Percent change in factor levels applied to all virtual patients post-exposure to levonorgestrel (lev). These values are the mean percent changes of the patient data reported in [17]

Coagulation factors	Increase after lev (%)
Factor II	12
Factor V	−3
Factor VII	12
Factor VIII	6
Factor X	22
