# Supplementary material for: Studying the Effects of Oral Contraceptives on Coagulation Using a Mathematical Modeling Approach
Source: Math Model Womens Health (2024). Author manuscript; Available in PMC 2025 Aug 19. (PMC12359859; doi:10.1007/978-3-031-58516-6_4)
Supplement: 1 [file NIHMS2100270-supplement-1.pdf]

Supplementary Information

The model includes the coagulation reactions shown in Fig. 12a. The reactions involve many coagulation proteins: inactive enzyme precursors (zymogens), active enzymes, and inactive and active cofactors. Active cofactors are not enzymes themselves but act to make the enzymes to which they are bound more effective than if they would be alone. In Fig. 12a, the zymogens are FVII, FIX, FX, FXI, and FII (prothrombin), which have respective active enzymes FVIIa, FIXa, FXa, FXIa, and FIIa (thrombin). The inactive/active cofactor pairs are FV/FVh/FVa and FVIII/FVIIIa. It is also shown that many of the coagulation reactions occur only on a cellular surface, some on the subendothelium (SE), some on the endothelium (EC), and others on an activated platelet's surface (APS). There are three critical surface-bound enzyme-cofactor complexes: TF:FVIIa on the SE ("extrinsic tenase"), plt-FVIIIa:FIXa ("intrinsic tenase," which we refer to simply as tenase), and plt-FVa:FXa ("prothrombinase") on an APS. Their substrates (i.e., the proteins that the enzyme complexes activate) must also be bound to the cellular surface to become activated [35].

The mathematical model simulates the clotting response due to a small injury to a vessel wall. The response is monitored in a reaction zone (RZ) above a region where tissue factor (TF) in the SE is exposed to flowing blood (Fig. 12b). Within the RZ, coagulation protein concentrations are assumed to change due to transport into and out of the RZ and due to their involvement in the coagulation reactions depicted in Fig. 12a. Similarly, platelet concentrations change as platelets adhere to the injured wall, become activated, and are transported into and out of the RZ. The height of the RZ and the rate of platelet and protein transport into and out of the RZ depend on the

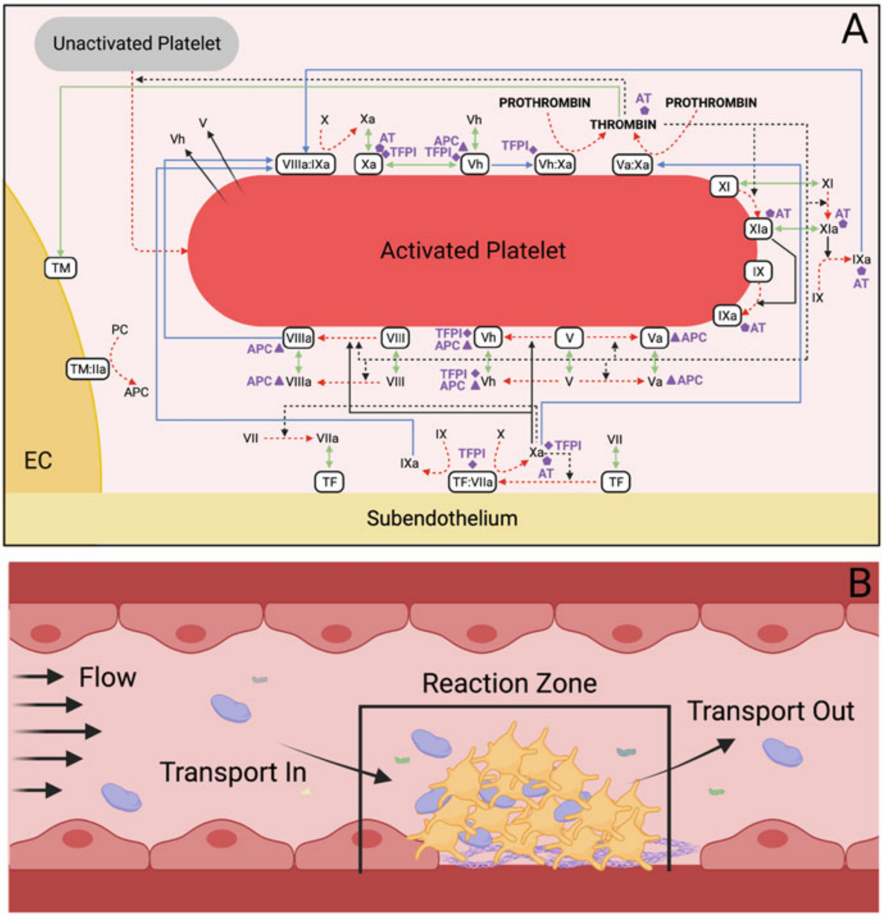

Fig. 12 Schematic of flow-mediated coagulation model. (**a**) Schematic of coagulation reactions included in the model. Dashed red arrows show cellular or chemical activation processes. Blue arrows show chemical transport in the fluid or on a surface. Green arrows depict binding and unbinding from cell surfaces. White boxes denote surface-bound species. Solid black lines show enzyme action in a forward direction, while dashed black lines show feedback action of enzymes. Black lines with a fade indicate release from the platelet. Purple shapes show inhibitors. (**b**) Schematic of the reaction zone. Notation: The lowercase letter "a" on any species means that it is in an "activated" form, e.g., FX and FXa are clotting factor X and activated clotting factor FX. EC: endothelial cell. See the text for other species definitions

flow's shear rate and on the species' diffusivities. Each species in the RZ is assumed to be uniformly distributed (well-mixed) and is described by its concentration, whose dynamics are tracked through an ordinary differential equation. Adjacent to the RZ, in the direction perpendicular to the flow, is an endothelial zone with height equal to that of the RZ and width dependent on the flow shear rate and protein diffusion coefficients [36]. Each species in the endothelial zone is also assumed to

be well-mixed. Endothelial cells also protrude into the RZ, and any reaction in the endothelial zone can also occur in the RZ.

Platelets are either (i) unactivated, unattached, and so free to move with the fluid or (ii) activated, bound to the SE or to other activated platelets (APs), and therefore stationary. Platelet activation occurs by contact with the SE, by exposure to thrombin, or by contact with other APs. The last of these is used as a surrogate for activation by platelet-released ADP, which we do not explicitly track in this model. Activation results in the release of platelet-derived FV with no FVa functionality or resistance to APC. Additionally, activation upregulates binding sites for coagulation proteins involved in surface-bound reactions. We characterize each coagulation protein not only by its chemical identity but also by whether it is in the fluid, bound to the SE or bound to an APS. Proteins bound to a surface are stationary, whereas proteins in the plasma move with the fluid. During a transition from SE to APS, or *vice versa*, a protein is subjected to flow and thus might be carried downstream.

An in-house FORTRAN program is used to set up the system of differential equations, set parameter values, and run the simulation. It uses the software package DLSODE [37] to solve the differential equations. Simulation sampling was carried out via a Python wrapper of the FORTRAN program. Graphical processing of simulation results was performed with MATLAB.

For each simulation, we specify the initial plasma concentrations of platelet and protein species, the rate constants for all reactions, the numbers of specific binding sites for coagulation factors on each APS, the dimensions of the injury, the flow velocity near the injured wall, the diffusion coefficients for all fluid-phase species, and the density of exposed TF. The outputs of the simulation are the concentration of every protein species in the RZ at each instant of time from initiation of the injury until the completion of the simulation and the concentrations of platelets attached either directly to the SE or to other platelets.

We have listed the full model equations for all species in Eqs. (4)–(122). Critical parameters are listed in Tables 2, 3, 4, 5, 6, 7, and 8. The model detailed includes extensions of our previous work [28–32]. New terms are in bold and underlined in Eqs. (23), (40), (121), and (122). The model consists of 119 species (and their corresponding ordinary differential equations) and 239 parameters including kinetic rates and initial/upstream concentrations. The solution of the model equations was carried out with our in-house FORTRAN code that uses DLSODE for the numerical solution of the differential equations; each run of the model that simulates 40 min of clotting activity takes less than 10 s on a Linux-based laptop. Simulations of this model (in the absence of heparin) can be performed with our online coagulation simulator ClotSims available at https://clotsims.app.

$$\begin{aligned}\frac{d}{dt}z_7 = &- k_7^{on} z_7 [TF]^{avail} + k_7^{off} z_7^m - k^+_{z_7:e_2} z_7 e_2 \\ &+ k^-_{z_7:e_2}[Z_7 : E_2] - k^+_{z_7:e_{10}} z_7 e_{10} + k^-_{z_7:e_{10}}[Z_7 : E_{10}] \\ &+ k_{flow}(z_7^{up} - z_7) - k^+_{z_7:e_9} z_7 e_9 + k^-_{z_7:e_9}[Z_7 : E_9]\end{aligned} \tag{4}$$

Table 2 Normal concentrations and surface binding site numbers

Species	Values and units	Notes
Prothrombin	1.4 μM	a
Factor V	0.01 μM	b
Factor VII	0.01 μM	a
Factor VIIa	0.1 nM	c
Factor VIII	1.0 nM	a
Factor IX	0.09 μM	a
Factor X	0.17 μM	a
Factor XI	30.0 nM	a
TFPI	0.5 nM	d
Protein C	65 nM	e
Platelet count	2.5×10^5 μl	e
N_2	1000/plt	f
N_2^*	1000/plt	f
N_5	3000/plt	g
N_8	450/plt	h
N_9	250/plt	i
N_9^*	250/plt	i
N_{10}	2700/plt	j
N_{11}	1500/plt	k
N_{11}^*	250/plt	k
n_5	3000/plt	l
p_{PLAS}	0.167 nM	m
AT	2.4 nM	n

(a) From [38]. (b) From [39]. (c) [40] suggests that normal plasma concentration of fVIIa is about 1% of the normal fVII concentration. (d) From [41]. (e) From [42]. (f) Estimated as described in the text of the supplementary information. (g) From [43]. (h) From [44]. (i) From [45]. (j) From [46]. (k) From [47, 48]. (l) Number of fV molecules released per activated platelet [49]. (m) Maximum concentration of platelets in a 2 μm high reaction zone assuming that 20 platelets can cover a 10 μm × 10 μm injured surface [50]. (n) From [51]

$$\begin{aligned}\frac{d}{dt}e_7 = &- k_7^{on} e_7 [TF]^{avail} + k_7^{off} e_7^m + k_{z_7:e_2}^{cat}[Z_7 : E_2] \\ &+ k_{z_7:e_{10}}^{cat}[Z_7 : E_{10}] + k_{flow}(e_7^{up} - e_7) + k_{z_7:e_9}^{cat}[Z_7 : E_9]\end{aligned} \tag{5}$$

$$\begin{aligned}\frac{d}{dt}z_{10} = &- k_{10}^{on} z_{10} p_{10}^{avail} + k_{10}^{off} z_{10}^m - k_{z_{10}:e_7^m}^{+} e_7^m \\ &+ k_{z_{10}:e_7^m}^{-}[Z_{10} : E_7^m] + k_{flow}(z_{10}^{up} - z_{10})\end{aligned} \tag{6}$$

Table 3 Binding to platelet surfaces

Reaction	Reactants	Products	($M^{-1}sec^{-1}$)	(sec^{-1})	Note
Factor IX	Z_9, P_9	Z_9^m	$k_9^{on} = 1.0 \times 10^7$	$k_9^{off} = 2.5 \times 10^{-2}$	a
Factor IXa	E_9, P_9	E_9^m	$k_9^{on} = 1.0 \times 10^7$	$k_9^{off} = 2.5 \times 10^{-2}$	a
Factor IXa	E_9, P_9^*	$E_9^{m,*}$	$k_9^{on} = 1.0 \times 10^7$	$k_9^{off} = 2.5 \times 10^{-2}$	b
Factor X	Z_{10}, P_{10}	Z_{10}^m	$k_{10}^{on} = 1.0 \times 10^7$	$k_{10}^{off} = 2.5 \times 10^{-2}$	a
Factor Xa	E_{10}, P_{10}	E_{10}^m	$k_{10}^{on} = 1.0 \times 10^7$	$k_{10}^{off} = 2.5 \times 10^{-2}$	a
Factor V	Z_5, P_5	Z_5^m	$k_5^{on} = 5.7 \times 10^7$	$k_5^{off} = 0.17$	c
Factor Vh	E_5^h, P_5	E_5^{hm}	$k_5^{on} = 5.7 \times 10^7$	$k_5^{off} = 0.17$	c
Factor Va	E_5, P_5	E_5^m	$k_5^{on} = 5.7 \times 10^7$	$k_5^{off} = 0.17$	c
Factor VIII	Z_8, P_8	Z_8^m	$k_8^{on} = 5.0 \times 10^7$	$k_8^{off} = 0.17$	d
Factor VIIIa	E_8, P_8	E_8^m	$k_8^{on} = 5.0 \times 10^7$	$k_8^{off} = 0.17$	d
Factor II	Z_2, P_2	Z_2^m	$k_2^{on} = 1.0 \times 10^7$	$k_2^{off} = 5.9$	e
Factor IIa	E_2, P_2	E_2^m	$k_2^{*,on} = 1.0 \times 10^7$	$k_2^{*,off} = 0.2$	f
Factor XI	Z_{11}, P_{11}	Z_{11}^m	$k_{z_{11}}^{on} = 1.0 \times 10^7$	$k_{z_{11}}^{off} = 0.1$	g
Factor XIa	E_{11}, P_{11}^*	E_{11}^m	$k_{e_{11}}^{on} = 1.0 \times 10^7$	$k_{e_{11}}^{off} = 0.017$	h

(a) For fIX binding to platelets, $K_d = 2.5 \times 10^{-9}$ M [45], and for fX binding to platelets, K_d has approximately the same value [43]. For fX binding to PCPS vesicles, the on-rate is about 10^7 $M^{-1}sec^{-1}$, and the off-rate is about 1.0 sec^{-1} [52] giving a dissociation constant of about 10^{-7} M. To estimate on- and off-rates for the higher affinity binding of fX to platelets, we keep the on-rate the same as for vesicles and adjust the off-rate to give the correct dissociation constant. The rates for fIX binding with platelets are taken to be the same as for fX binding. (b) We assume binding constants for fIXa binding to the specific fIXa binding sites are the same as for shared sites. (c) fV binds with high affinity to phospholipids (PCPS) [52], and we use the same rate constants reported there to describe fV binding to platelets. (d) The K_d for fVIII binding with platelets is taken from [44]. We set the off-rate k_8^{off} for fVIII binding to platelets equal to that for fV binding to platelets and calculate the on-rate k_8^{on}. (e) For prothrombin interactions with platelets, K_d is reported to be 5.9×10^{-7} M [53]. We choose k_2^{off} and set $k_2^{on} = k_2^{off}/K_d$. (f) Estimated as described in the text of the supplementary information. (g) K_d = 10 nM [54]. (h) K_d = 1.7 nM [48]

$$\begin{aligned}\frac{d}{dt}e_{10} = &- k_{10}^{on}e_{10}p_{10}^{avail} + k_{10}^{off}e_{10}^m + k_{z_{10}:e_7^m}^{cat}[Z_{10}:E_7^m] \\ &+ (k_{z_7:e_{10}}^{cat} + k_{z_7:e_{10}}^{-})[Z_7:E_{10}] - k_{z_7:e_{10}}^{+}e_{10}z_7 \\ &+ (k_{z_7^m:e_{10}}^{cat} + k_{z_7^m:e_{10}}^{-})[Z_7^m:E_{10}] - k_{z_7^m:e_{10}}^{+}e_{10}z_7^m \\ &- k_{TFPI:e_{10}}^{+}e_{10}[TFPI] + k_{TFPI:e_{10}^m}^{-}[TFPI:E_{10}]\end{aligned} \quad (7)$$

Table 4 Reactions on subendothelium. Notation: activation (of -, by -); binding (of -, with -)

Reaction	Reactants	Complex	Product	$(M^{-1}sec^{-1})$	(sec^{-1})	(sec^{-1})	Notes
Activation							
(TF:VII,fXa)	E_{10}, Z_7^m	$Z_7^m : E_{10}$	E_7^m	$k^+_{z_7^m:e_{10}} = 5.0 \times 10^6$	$k^-_{z_7^m:e_{10}} = 1.0$	$k^{cat}_{z_7^m:e_{10}} = 5.0$	a
(TF:VII, fIIa)	E_2, Z_7^m	$Z_7^m : E_2$	E_7^m	$k^+_{z_7^m:e_2} = 3.92 \times 10^5$	$k^-_{z_7^m:e_2} = 1.0$	$k^{cat}_{z_7^m:e_2} = 6.1 \times 10^{-2}$	b
(fX, TF:VIIa)	E_7^m, Z_{10}	$Z_{10} : E_7^m$	E_{10}	$k^+_{z_{10}:e_7^m} = 5.0 \times 10^6$	$k^-_{z_{10}:e_7^m} = 1.0$	$k^{cat}_{z_{10}:e_7^m} = 1.15$	c
(fIX, TF:VIIa)	E_7^m, Z_9	$Z_9 : E_7^m$	E_9	$k^+_{z_9:e_7^m} = 9.4 \times 10^6$	$k^-_{z_9:e_7^m} = 1.0$	$k^{cat}_{z_9:e_7^m} = 1.15$	d
Binding							
(fVII, TF)	Z_7, TF		Z_7^m	$k_7^{on} = 5.0 \times 10^7$	$k_7^{off} = 5.0 \times 10^{-3}$		e
(fVIIa, TF)	E_7, TF		E_7^m	$k_7^{on} = 5.0 \times 10^7$	$k_7^{off} = 5.0 \times 10^{-3}$		e

(a) $K_M = 1.2 \times 10^{-6}$ M [55]. (b) $K_M = 2.7 \times 10^{-6}$ M [55]. (c) $K_M = 4.5 \times 10^{-7}$ M [38]. (d) $K_M = 2.4 \times 10^{-7}$ M [56]. (e) $K_d = 1.0 \times 10^{-10}$ M [57]

Table 5 Reactions in the plasma. All of the reactions are activation reactions. Notation: activation (of -, by -)

Reaction	Reactants	Complex	Product	$(M^{-1}sec^{-1})$	(sec^{-1})	(sec^{-1})	Note
(fVII, fXa)	Z_7, E_{10}	$Z_7 : E_{10}$	E_7	$k^+_{z_7:e_{10}} = 5 \times 10^6$	$k^-_{z_7:e_{10}} = 1.0$	$k^{cat}_{z_7:e_{10}} = 5.0$	a
(fVII, fIIa)	Z_7, E_2	$Z_7 : E_2$	E_7	$k^+_{z_7:e_2} = 3.92 \times 10^5$	$k^-_{z_7:e_2} = 1.0$	$k^{cat}_{z_7:e_2} = 6.1 \times 10^{-2}$	b
(fV, fIIa)	Z_5, E_2	$Z_5 : E_2$	E_5	$k^+_{z_5:e_2} = 1.73 \times 10^7$	$k^-_{z_5:e_2} = 1.0$	$k^{cat}_{z_5:e_2} = 0.23$	c
(fVIII, fIIa)	Z_8, E_2	$Z_8 : E_2$	E_8	$k^+_{z_8:e_2} = 2.64 \times 10^7$	$k^-_{z_8:e_2} = 1.0$	$k^{cat}_{z_8:e_2} = 0.9$	d
(fXI-fXI, fIIa)	Z_{11}, E_2	$Z_{11} : E_2$	E^h_{11}	$k^+_{z_{11}:e_2} = 2.0 \times 10^7$	$k^-_{z_{11}:e_2} = 1.0$	$k^{cat}_{z_{11}:e_2} = 1.3 \times 10^{-4}$	e
(fIX, fXIa)	Z_9, E^h_{11}	$Z_9 : E^h_{11}$	E_9	$k^+_{z_9:e^h_{11}} = 0.6 \times (10)^7$	$k^-_{z_9:e^h_{11}} = 1.0$	$k^{cat}_{z_9:e^h_{11}} = 0.21$	f

(a) $K_M = 1.2 \times 10^{-6}$ M [55]. (b) $K_M = 2.7 \times 10^{-6}$ M [55] (c) $K_M = 7.17 \times 10^{-8}$ M [58]. (d) [59], $K_M = 2 \times 10^{-7}$ M [60]. (e) $K_M = 50$ nM [61]. Rate constants apply also for thrombin activation of XIa-XI. (f) $K_M = 0.2\,\mu$M [62, 63] and for activation of IX by XIa-XIa

Table 6 Platelet transitions

Reactants	Reactants	Products	($M^{-1}sec^{-1}$)	(sec^{-1})	Note
Unactivated platelet adhering to SE	PL, SE	PL_a^s	$k_{\text{adh}}^{+} = 2 \times 10^{10}$	$k_{\text{adh}}^{-} = 0$	a
Activated platelet adhering to SE	PL_a^v, SE	PL_a^v	$k_{\text{adh}}^{+} = 2 \times 10^{10}$	$k_{\text{adh}}^{-} = 0$	a
Platelet activation by platelet in solution	PL, PL_a^v	$2PL_a^v$	$k_{plt}^{\text{act}} = 3 \times 10^8$		b
Platelet activation on SE	PL, PL_a^s	PL_a^v, PL_a^s	$k_{plt}^{\text{act}} = 3 \times 10^8$		b
Platelet activation by thrombin	PL, E_2	PL_a^v		$k_{e_2}^{\text{act}} = 0.50$	b

(a) Estimated from data in [64, 65] as described in [28]. (b) Estimated from data in [66] as described in [28]. SE = subendothelium

Table 7 Reactions on platelet surfaces. Notation: activation (of -, by -); binding (of -, with -)

Reaction	Reactants	Complex	Product	($M^{-1}sec^{-1}$)	(sec^{-1})	(sec^{-1})	Note
Activation							
(V, Xa)	Z_5^m, E_{10}^m	$Z_5^m : E_{10}^m$	E_5^{hm}	$k^+_{z_5^m:e_{10}^m} = 1.0 \times 10^8$	$k^-_{z_5^m:e_{10}^m} = 1.0$	$k^{cat}_{z_5^m:e_{10}^m} = 4.6 \times 10^{-2}$	a
(V, IIa)	Z_5^m, E_2^m	$Z_5^m : E_2^m$	E_5^m	$k^+_{z_5^m:e_2^m} = 1.73 \times 10^7$	$k^-_{z_5^m:e_2^m} = 1.0$	$k^{cat}_{z_5^m:e_2^m} = 0.23$	b
(Vh, IIa)	E_5^{hm}, E_2^m	$E_5^{hm} : E_2^m$	E_5^m	$k^+_{z_5^m:e_2^m} = 1.73 \times 10^7$	$k^-_{z_5^m:e_2^m} = 1.0$	$k^{cat}_{z_5^m:e_2^m} = 0.23$	b
(VIII, Xa)	Z_8^m, E_{10}^m	$Z_8^m : E_{10}^m$	E_8^m	$k^+_{z_8^m:e_{10}^m} = 5.1 \times 10^7$	$k^-_{z_8^m:e_{10}^m} = 1.0$	$k^{cat}_{z_8^m:e_{10}^m} = 2.3 \times 10^{-2}$	c
(VIII, IIa)	Z_8^m, E_2^m	$Z_8^m : E_2^m$	E_8^m	$k^+_{z_8^m:e_2^m} = 2.64 \times 10^7$	$k^-_{z_8^m:e_2^m} = 1.0$	$k^{cat}_{z_8^m:e_2^m} = 0.9$	d
(X, VIIIa:IXa)	Z_{10}^m, TEN	$Z_{10}^m : TEN$	E_{10}^m	$k^+_{z_{10}^m:ten} = 1.31 \times 10^8$	$k^-_{z_{10}^m:ten} = 1.0$	$k^{cat}_{z_{10}^m:ten} = 20.0$	f
(X, VIIIa:IXa*)	Z_{10}^m, TEN^*	$Z_{10}^m : TEN^*$	E_{10}^m	$k^+_{z_{10}^m:ten} = 1.31 \times 10^8$	$k^-_{z_{10}^m:ten} = 1.0$	$k^{cat}_{z_{10}^m:ten} = 20.0$	f
(II, Vh:Xa)	$Z_2^m, PROh$	$Z_2^m : PROh$	E_2^m	$k^+_{z_2^m:pro} = 1.03 \times 10^8$	$k^-_{z_2^m:pro} = 1.0$	$k^{cat}_{z_2^m:pro} = 30.0$	g
(II, Va:Xa)	Z_2^m, PRO	$Z_2^m : PRO$	E_2^m	$k^+_{z_2^m:pro} = 1.03 \times 10^8$	$k^-_{z_2^m:pro} = 1.0$	$k^{cat}_{z_2^m:pro} = 30.0$	g
(XI-XI, IIa)	Z_{11}^m, E_2^m	$Z_{11}^m : E_2^m$	E_{11}^{hm}	$k^+_{z_{11}^m:e_2^m} = 2.0 \times 10^7$	$k^-_{z_{11}^m:e_2^m} = 1.0$	$k^{cat}_{z_{11}^m:e_2^m} = 1.3 \times 10^{-4}$	h
(IX, XIa)	Z_9^m, E_{11}^{hm}	$Z_9^m : E_{11}^{hm}$	E_9	$k^+_{z_9^m:e_{11}^m} = 0.6 \times 10^7$	$k^-_{z_9^m:e_{11}^m} = 1.0$	$k^{cat}_{z_9^m:e_{11}^m} = 0.21$	i

(continued)

Table 7 (continued)

Reaction	Reactants	Complex	Product	($M^{-1}sec^{-1}$)	(sec^{-1})	(sec^{-1})	Note
Binding							
(VIIIa, IXa)	E_8^m, E_9^m		TEN	$k_{ten}^{+} = 1.0 \times 10^8$	$k_{ten}^{-} = 0.01$		e
(VIIIa, IXa*)	$E_8^m, E_9^{m,*}$		TEN^*	$k_{ten}^{+} = 1.0 \times 10^8$	$k_{ten}^{-} = 0.01$		e
(Vh, Xa)	E_5^{hm}, E_{10}^m		$PROh$	$k_{pro}^{+} = 1.0 \times 10^8$	$k_{pro}^{-} = 0.01$		e
(Va, Xa)	E_5^m, E_{10}^m		PRO	$k_{pro}^{+} = 1.0 \times 10^8$	$k_{pro}^{-} = 0.01$		e

(a) $K_M = 10.4 \times 10^{-9}$ M [67]. (b) The rate constants for thrombin activation of fV on platelets are assumed to be the same as in plasma. (c) $K_M = 2.0 \times 10^{-8}$ M [60]. (d) The rate constants for thrombin activation of fVIII on platelets are assumed to be the same as in plasma. (e) The formation of the tenase and prothrombinase complexes is assumed to be very fast with $K_d = 1.0 \times 10^{-10}$ M [68]. (f) $K_M = 1.6 \times 10^{-7}$ M [69]. (g) $K_M = 3.0 \times 10^{-7}$ M [70]. (h) $K_M = 50$ nM [61]. Rate constants apply also for thrombin activation of Plt-XIa-XI. (i) $K_M = 0.2\,\mu$M [62, 63]. Rate constants apply also for activation of platelet-bound IX by Plt-XIa-XIa

Table 8 Inhibition reactions. Notation: inactivation (of -, by -); activation (of -, by -); binding (of -, with -)

Reaction	Reactants	Product	($M^{-1}sec^{-1}$)	(sec^{-1})	(sec^{-1})	Note
Inactivation						
(IXa, AT-III)	E_9, AT	$E_9 : AT$		$k_{e_9}^{AT} = 4.8 \times 10^2$		a
(Xa, AT-III)	E_{10}, AT	$E_{10} : AT$		$k_{e_{10}}^{AT} = 3.5 \times 10^3$		a
(IIa, AT-III)	E_2, AT	$E_2 : AT$		$k_{e_2}^{AT} = 1.4 \times 10^4$		a
(XIa, AT-III)	E_{11}, AT	$E_{11} : AT$		$k_{e_{11}}^{AT} = 2.4 \times 10^2$		a
(XIa:AT, AT-III)	$E_{11} : AT$, AT	$AT : E_{11} : AT$		$k_{e_{11}}^{AT} = 2.4 \times 10^2$		a
(APC, Va)	$APC,\ E_5^m$	None	$k_{e_5^m:APC}^{+} = 1.2 \times 10^8$	$k_{e_5^m:APC}^{-} = 1.0$	$k_{e_5^m:APC}^{\text{cat}} = 0.5$	c
(APC, VIIIa)	$APC,\ E_8^m$	None	$k_{e_8^m:APC}^{+} = 1.2 \times 10^8$	$k_{e_8^m:APC}^{-} = 1.0$	$k_{e_8^m:APC}^{\text{cat}} = 0.5$	c
Binding						
(TFPI, Xa)	$TFPI, E_{10}$	$TFPIa$	$k_{tfpia:e_{10}}^{+} = 1.6 \times 10^7$	$k_{tfpia:e_{10}}^{-} = 3.3 \times 10^{-4}$		d
(TFPI, Vh)	TFPI, E_5^h	$TFPI : E_5^h$	$k_{tfpi:e5h}^{+} = 0.05 \times 10^9$	$k_{tfpi:e5h}^{-} = 0.0045$		e
(TFPI:Xa, Vh)	TFPIa, E_5^h	$E_5^h : TFPI : E_{10}$	$k_{tfpi:e5h}^{+} = 0.05 \times 10^9$	$k_{tfpi:e5h}^{-} = 0.0045$		e
(TFPI:Vh, Xa)	$TFPI : E_5^h, E_{10}$	$E_5^h : TFPI : E_{10}$	$k_{tfpia:e_{10}}^{+} = 1.6 \times 10^7$	$k_{tfpia:e_{10}}^{-} = 3.3 \times 10^{-4}$		d

(continued)

Table 8 (continued)

Reaction	Reactants	Product	($M^{-1}sec^{-1}$)	(sec^{-1})	(sec^{-1})	Note
(Xa:Vh, TFPI)	$E_{10} : E_5^h, TFPIa$	$TFPI : E_{10} : E_5^h$	$k^+_{tfpibprohv10} = 1.6 \times 10^7$	$k^-_{tfpibprohv10} = 3.3 \times 10^-4$		d
(TFPIa, TF:VIIa)	$TFPIa, E_7^m$	$TFPIa : E_7^m$	$k^+_{tfpia:e_{10}} = 1.6 \times 10^7$	$k^-_{tfpia:e_{10}} = 3.3 \times 10^{-4}$		f
(TM, Thrombin)	$TM,\ E_2^{ec}$	$TM : E_2^{ec}$	$k^{\text{on}}_{TM} = 1.0 \times 10^8$	$k^{\text{off}}_{TM} = 5.0 \times 10^{-2}$		g
(TM_{RZ}, Thrombin)	$TM_{RZ},\ E_2$	$TM_{RZ} : E_2$	$k^{\text{on}}_{TM_{RZ}} = 1.0 \times 10^8$	$k^{\text{off}}_{TM_{RZ}} = 5.0 \times 10^{-2}$		g
Activation						
(PC, $TM_{RZ} : E_2$)	$PC, TM_{RZ} : E_2$	APC	$k^+_{pc} = 1.7 \times 10^6$	$k^-_{pc} = 1.0$	$k^{cat}_{pc} = 0.16$	i
(PC, TM:E_2^{ec})	$PC, TM : E_2^{ec}$	APC	$k^+_{PC:TM:e_2^{ec}} = 1.7 \times 10^6$	$k^-_{PC:TM:e_2^{ec}} = 1.0$	$k^{\text{cat}}_{PC:TM:e_2^{ec}} = 0.16$	i

(a) From [51]. (b) From [71]. (c) For inhibition of fVa by APC, $K_M = 12.5 \times 10^{-9}$ [72]. We assume the same reaction rates for the inhibition of fVIIIa by APC. (d) From [73]. (e) From [74]. (f) From [75]. (g) $K_d = 0.5$ nM and $[PC] = 65$ nM [76]. (h) From [77]. (i) $k_{PC:TM:e_2^{ec}} = 0.167\ sec^{-1}$, $K_M = 0.7 \times 10^{-6}$ M [78]

$$+ k_{flow}(e_{10}^{up} - e_{10}) - k_{diff}(e_{10} - e_{10}^{ec})$$
$$- k^{+}_{TFPI:e_5^h:e_{10}}[TFPI : E_5^h]e_{10}$$
$$- k^{-}_{TFPI:e_5^h:e_{10}}[E_{10} : TFPI : E_5^h]$$
$$- k^{+}_{TFPI:e_5^{hm}:e_{10}}[TFPI : E_5^{hm}]e_{10}$$
$$+ k^{-}_{TFPI:e_5^{hm}:e_{10}}[E_{10} : TFPI : E_5^{hm}]$$

$$\frac{d}{dt}z_{10}^m = k_{10}^{on} z_{10} p_{10}^{avail} - k_{10}^{off} z_{10}^m + k^{+}_{z_{10}^m:TEN} z_{10}^m [TEN] \tag{8}$$
$$+ k^{-}_{z_{10}^m:TEN}[Z_{10}^m : TEN] - k^{+}_{z_{10}^m:TEN} z_{10}^m [TEN^*]$$
$$+ k^{-}_{z_{10}^m:TEN}[Z_{10}^m : TEN^*]$$

$$\frac{d}{dt}e_{10}^m = k_{10}^{on} e_{10} p_{10}^{avail} - k_{10}^{off} e_{10}^m + k^{cat}_{z_{10}^m:TEN}[Z_{10}^m : TEN] \tag{9}$$
$$+ (k^{cat}_{z_5^m:e_{10}^m} + k^{-}_{z_5^m:e_{10}^m})[Z_5^m : E_{10}^m] - k^{+}_{z_5^m:e_{10}^m} e_{10}^m z_5^m$$
$$+ (k^{cat}_{z_8^m e_{10}^m} + k^{-}_{z_8^m e_{10}^m})[Z_8^m : E_{10^m}] - k^{+}_{z_8^m:e_{10}^m} e_{10}^m z_8^m$$
$$+ k^{-}_{e_5^m:e_{10}^m}[PRO] - k^{p}_{e_5^m:e_{10}^m} e_{10}^m e_5^m$$
$$+ k^{cat}_{z_{10}^m:TEN}[Z_{10}^m : TEN^*] - k^{+}_{e_5^{hm}:e_{10}^m} e_{10}^m e_5^{hm}$$
$$+ k^{-}_{e_5^{hm}:e_{10}^m} PRO^h - k^{+}_{TFPI:e_{10}^m} e_{10}^m TFPI$$
$$+ k^{-}_{TFPI:e_{10}^m}[TFPI : E_{10}^m]$$
$$- k^{+}_{TFPI:e_5^{hm}:e_{10}^m}[TFPI : E_5^{hm}]e_{10}^m$$
$$+ k^{-}_{TFPI:e_5^{hm}:e_{10}^m}[E_{10}^m : TFPI : E_5^{hm}]$$
$$- k^{+}_{TFPI:e_{10}:e_5^{hm}}[TFPI : E_5^{hm}]e_{10}^m$$
$$+ k^{-}_{TFPI:e_{10}:e_5^{hm}}[TFPI : PRO_{v5}^h] - k^{AT}_{e_{10}^m} e_{10}^m [AT]$$

$$\frac{d}{dt}z_5 = - k_5^{on} z_5 p_5^{avail} + k_5^{off} z_5^m - k^{+}_{z_5:e_2} z_5 e_2 \tag{10}$$
$$+ k^{-}_{z_5:e_2}[Z_5 : E_2] + k_{flow}(z_5^{up} - z_5)$$
$$+ n_5(k^{+}_{adh} p^{avail}_{PLAS} + k^{act}_{plt}([PL_a^v] + [PL_a^s])$$
$$+ k^{act}_{e2}\frac{e_2}{e_2 + 0.001})[PL]$$

$$\frac{d}{dt}e_5 = -k_5^{on}e_5 p_5^{avail} + k_5^{off}e_5^m + k_{z_5:e_2}^{cat}[Z_5 : E_2] + k_{flow}(e_5^{up} - e_5) + k_{e_5:APC}^{-}[APC : E_5] - k_{e_5:APC}^{+}e_5[APC] + k_{e_5^h:e_2}^{cat}[E_5^h : E_2] \tag{11}$$

$$\frac{d}{dt}z_5^m = k_5^{on}z_5 p_5^{avail} - k_5^{off}z_5^m - k_{z_5^m e_{10}^m}^{+}z_5^m e_{10}^m + k_{z_5^m:e_{10}^m}^{-}[Z_5^m : E_{10}^m] - k_{z_5^m:e_2^m}^{+}z_5^m e_2^m + k_{z_5^m e_2^m}^{-}[Z_5^m : E_2^m] \tag{12}$$

$$\frac{d}{dt}e_5^m = k_5^{on}e_5 p_5^{avail} - k_5^{off}e_5^m + k_{z_5^m:e_2^m}^{cat}[Z_5^m : E_2^m] + k_{e_5^m:APC}^{-}[APC : E_5^m] - k_{e_5^m:APC}^{+}e_5^m[APC] - k_{e_5^m:e_{10}^m}^{+}e_5^m e_{10}^m + k_{e_5^m:e_{10}^m}^{-}[PRO] + k_{e_5^{hm}:e_2^m}^{cat}[E_5^{hm} : E_2^m] \tag{13}$$

$$\frac{d}{dt}z_8 = -k_8^{on}z_8 p_8^{avail} + k_8^{off}z_8^m + k_{flow}(z_8^{up} - z_8) - k_{z_8:e_2}^{+}z_8 e_2 + k_{z_8:e_2}^{-}[Z_8 : E_2] \tag{14}$$

$$\frac{d}{dt}e_8 = -k_8^{on}e_8 p_8^{avail} + k_8^{off}e_8^m + k_{flow}(e_8^{up} - e_8) + k_{z_8:e_2}^{cat} - 0.005e_8 + k_{e_8:APC}^{-}[APC : E_8] - k_{e_8:APC}^{+}e_8[APC] \tag{15}$$

$$\frac{d}{dt}z_8^m = k_8^{on}z_8 p_8^{avail} - k_8^{off}z_8^m - k_{z_8^m:e_{10}^m}^{+}z_8^m e_{10}^m + k_{z_8^m:e_{10}^m}^{-}[Z_8^m : E_{10}^m] - k_{z_8^m:e_2^m}^{+}z_8^m e_2^m + k_{z_8^m:e_2^m}^{-}[Z_8^m : E_2^m] \tag{16}$$

$$\frac{d}{dt}e_8^m = k_8^{on}e_8 p_8^{avail} - k_8^{off}e_8^m + k_{z_8^m:e_{10}^m}^{cat}[Z_8^m : E_{10}^m] + k_{z_8^m:e_2^m}^{cat}[Z_8^m : E_2^m] - k_{e_8^m:APC}^{+}e_8^m[APC] + k_{e_8^m:APC}^{-}[APC : E_8^m] - k_{e_8^m:e_9^m}^{+}e_9^m e_8^m - 0.005e_8^m + k_{e_8^m:e_9^m}^{-}[TEN] - k_{e_8^m:e_9^m}^{+}e_8^m e_9^m + k_{e_8^m:e_9^m}^{-}[TEN] \tag{17}$$

$$\frac{d}{dt}z_9 = k_{flow}(z_9^{up} - z_9) - k_9^{on}p_9^{avail}z_9 + k_9^{off}z_9^m - k_{z_9:e_7^m}^{+}z_9 e_7^m + k_{z_9:e_7^m}^{-}[Z_9 : E_7^m] - k_{z_9:e_{11}^h}^{+}e_{11}^h + k_{z_9:e_{11}^h}^{-}[Z_9 : E_{11}^h] - k_{z_9:e_11}^{+}z_9 e_{11} + k_{z_9:e_{11}}^{-}[Z_9 : E_{11}] \tag{18}$$

$$\frac{d}{dt}e_9 = k_{flow}(e_9^{up} - e_9) - k_9^{on} p_9^{avail} e_9 + k_9^{off} e_9^m \quad (19)$$
$$+ k_{z_9:e_7^m}^{cat}[Z_9 : E_7^m] - k_{z_7:e_9}^{+} z_7 e_9$$
$$+ (k_{z_7:e_9}^{cat} + k_{z_7:e_9}^{-})[Z_7 : E_9]$$
$$+ (k_{z_7^m:e_9}^{cat} + k_{z_7^m:e_9}^{-})[Z_7^m : E_9] - k_{z_7^m:e_9}^{+} z_7^m e_9$$
$$- k_9^{on} p_9^{*,avail} e_9 + k_9^{off} e_9^{m*} - k_{diff}(e_9 - e_9^{ec})$$
$$+ k_{z_9:e_{11}^h}^{cat}[Z_9 : E_{11}^h] + k_{z_9:e_{11}}^{cat}[Z_9 : E_{11}] - k_{e_9}^{AT} e_9[AT]$$

$$\frac{d}{dt}z_9^m = k_9^{on} p_9^{avail} z_9 - k_9^{off} z_9^m - k_{z_9^m:e_{11}^{h,m}}^{+} z_9^m e_{11}^{h,m} \quad (20)$$
$$+ k_{z_9^m e_{11}^{h,m}}^{-}[Z_9^m : E_{11}^{h,m}] - k_{z_9^m:e_{11}^{m*}}^{+} z_9^m e_{11}^{m*}$$
$$+ k_{z_9^m e_{11}^{m*}}^{-}[Z_9^m : E_{11}^{m*}]$$

$$\frac{d}{dt}e_9^m = k_9^{on} p_9^{avail} e_9 - k_9^{off} e_9^m - k_{e_8^m:e_9^m}^{+} e_8^m e_9^m \quad (21)$$
$$+ k_{e_8^m:e_9^m}^{-}[TEN] + k_{z_9^m:e_{11}^{h,m}}^{cat}[Z_9^m : E_{11}^{h,m}]$$
$$+ k_{z_9^m:e_{11}^{m*}}^{cat}[Z_9^m : E_{11}^{m*}] - k_{e_9^m}^{AT} e_9^m[AT]$$

$$\frac{d}{dt}z_2 = - k_2^{on} p_2^{avail} z_2 + k_2^{off} z_2^m + k_{flow}(z_2^{up} - z_2) \quad (22)$$

$$\frac{d}{dt}e_2 = k_{flow}(e_2^{up} - e_2) - k_{2*}^{on} p_2^{*,avail} e_2 + k_{2*}^{off} e_2^m \quad (23)$$
$$+ k_{z_2^m:PRO}^{cat}[Z_2^m : PRO] - k_{z_5:e_2^p} z_5 e_2$$
$$+ (k_{z_5:e_2}^{cat} + k_{z_5:e_2}^{-})[Z_5 : E_2] - k_{z_8:e_2}^{+} z_8 e_2$$
$$+ (k_{z_8:e_2}^{cat} + k_{z_8:e_2}^{-})[Z_8 : E_2] - k_{z_7:e_2}^{+} z_7 e_2$$
$$+ (k_{z_7^m:e_2}^{cat} + k_{z_7:e_2}^{-})[Z_7 : E_2] - k_{z_7^m e_2}^{+} z_7^m e_2$$
$$+ (k_{z_7^m:e_2}^{cat} + k_{z_7^m:e_2}^{-})[Z_7^m : E_2] - k_{diff}(e_2 - e_2^{ec})$$
$$- k_{z_{11}:e_2}^{+} z_{11} + (k_{z_{11}:e_2}^{-} + k_{z_{11}:e_2}^{cat})[Z_{11} : E_2]$$
$$- k_{e_{11}^h:e_2}^{+} e_{11}^h e_2 + (k_{e_{11}^h:e_2}^{-} + k_{e_{11}^h} : e_2)^{cat}[E_{11}^h : E_2]$$
$$+ k_{z_2^m:PRO^h}^{cat}[Z_2^m : PRO^h] - k_{e_5^h:e_2}^{+} e_2 e_5^h$$

$$+ k^{-}_{e_5^h:e_2}[E_5^h : E_2] + k^{cat}_{e_5^h:e_2}[E_5^h : E_2] - k^{AT}_{e_2} e_2[AT]$$

$$\underline{- \boldsymbol{k^{\text{on}}_{TM_{RZ}} e_2 [TM^{avail}_{RZ}] + k^{\text{off}}_{TM_{RZ}} [TM_{RZ} : E_2]}}$$

$$\frac{d}{dt} z_2^m = k_2^{on} p_2^{avail} z_2 - k_2^{off} z_2^m - k^{+}_{z_2^m:PRO} z_2^m [PRO] \tag{24}$$

$$+ k^{-}_{z_2^m:PRO}[Z_2^m : PRO] - k^{+}_{z_2^m:PRO^h} z_2^m PRO^h$$

$$+ k^{-}_{z_2^m:PRO^h}[Z_2^m : PRO^h]$$

$$\frac{d}{dt} e_2^m = k_{2*}^{on} p_2^{avail} e_2 - k_{2*}^{off} e_2^m \tag{25}$$

$$+ (k^{cat}_{z_5^m:e_2^m} + k^{-}_{z_5^m:e_2^m})[Z_5^m : E_2^m] - k^{+}_{z_5^m:e_2^m} z_5^m e_2^m$$

$$+ (k^{cat}_{z_8^m:e_2^m} + k^{-}_{z_8^m:e_2^m})[Z_8^m : E_2^m] - k^{+}_{z_8^m:E_2^m} z_8^m e_2^m$$

$$- k^{+}_{z_{11}^m:e_2^m} z_{11}^m e_2^m - k^{+}_{e_{11}^{h,m*} e_2^m} e_{11}^{h,m*} e_2^m$$

$$+ (k^{-}_{z_{11}^m:e_2^m} + k^{cat}_{z_{11}^m:e_2^m})[Z_{11}^m : E_2^m]$$

$$+ (k^{-}_{e_{11}^{h,m*} e_2^m} + k^{cat}_{e_{11}^{h,m*}:e_2^m})[E_{11}^{hms} : E_2^m]$$

$$- k^{+}_{e_5^{hm}:e_2^m} e_2^m e_5^{hm} + k^{-}_{e_5^{hm}:e_2^m}[E_5^{hm} : E_2^m]$$

$$+ k^{cat}_{e_5^{hm}:e_2^m}[E_5^{hm} : E_2^m] - k^{+}_{PRO^h:e_2^m} PRO^h e_2^m$$

$$+ k^{-}_{PRO^h:e_2^m}[PRO^h : E_2^m]$$

$$+ k^{cat}_{PRO^h:e_2^m}[PRO^h : E_2^m] - k^{AT}_{e_2^m} e_2^m [AT]$$

$$\frac{d}{dt}[TEN] = - k^{-}_{e_8^m:e_9^m}[TEN] + k_{e_8^m:e_9^m} e_8^m e_9^m \tag{26}$$

$$+ (k^{cat}_{z_{10}^m:TEN} + k^{-}_{z_{10}^m:TEN})[Z_{10}^m : TEN]$$

$$- k^{+}_{z_{10}^m:TEN} z_{10}^m [TEN]$$

$$\frac{d}{dt}[PRO] = - k^{-}_{e_5^m:e_{10}^m}[PRO] + k^{+}_{e_5^m:e_{10}^m} e_{10}^m e_5^m \tag{27}$$

$$+ (k^{cat}_{z_2^m:PRO} + k^{-}_{z_2^m:PRO})[Z_2^m : PRO]$$

$$- k^{+}_{z_2^m:PRO} z_2^m [PRO] + k^{cat}_{PRO^h:e_2^m}[PRO^h : E_2^m]$$

$$\frac{d}{dt}[PL_a^s] = k^{+}_{adh} p^{avail}_{PLAS}[PL] - k^{-}_{adh}[PL_a^s] + k^{+}_{adh}[PL_a^v] p^{avail}_{PLAS} \tag{28}$$

$$\frac{d}{dt}[PL] = k^{p}_{flow}([PL]^{up} - [PL]) - k^{+}_{adh} p^{avail}_{PLAS} + (k^{act}_{plt}([PL^{v}_{a}] + [PL^{s}_{a}]) + k^{act}_{e2}\frac{e_2}{e_2 + 0.001})[PL] \quad (29)$$

$$\frac{d}{dt}[PL^{v}_{a}] = k^{-}_{adh}[PL^{s}_{a}] - k^{+}_{adh}[PL^{v}_{a}] p^{avail}_{PLAS} + (k^{act}_{plt}([PL^{v}_{a}] + [PL^{s}_{a}]) + k^{act}_{e2}\frac{e_2}{e_2 + 0.001})[PL] \quad (30)$$

$$\begin{aligned}\frac{d}{dt} z^{m}_{7} = {} & k^{on}_{7} z_7 [TF]^{avail} - k^{off}_{7} z^{m}_{7} - k^{+}_{z^{m}_{7}:e_{10}} z^{m}_{7} e_{10} \\ & - k^{+}_{z^{m}_{7}:e_2} z^{m}_{7} e_2 + k^{-}_{z^{m}_{7}:e_{10}} [Z^{m}_{7} : E_{10}] \\ & + k^{-}_{z^{m}_{7}:e_2} [Z^{m}_{7} : E_2] - k^{+}_{z^{m}_{7}:e_9} z^{m}_{7} e_9 \\ & + k^{-}_{z^{m}_{7}:e_9} [Z^{m}_{7} : E_9] - z^{m}_{7} \frac{d}{dt}[PL^{s}_{a}] \frac{1}{p^{avail}_{PLAS}}\end{aligned} \quad (31)$$

$$\begin{aligned}\frac{d}{dt} e^{m}_{7} = {} & k^{on}_{7} e_7 [TF]^{avail} + k^{-}_{TFPI:e_{10}:e^{m}_{7}} [TFPI : E^{m}_{7}] \\ & - k^{off}_{7} e^{m}_{7} k^{+}_{TFPI:e_{10}:e^{m}_{7}} e^{m}_{7} [TFPI : E_{10}] \\ & + k^{cat}_{z^{m}_{7}:e_{10}} [Z^{m}_{7} : E_{10}] + k^{cat}_{z^{m}_{7}:e_2} [Z^{m}_{7} : E_2] \\ & + (k^{cat}_{z_{10}:e^{m}_{7}} + k^{-}_{z_{10}:e^{m}_{7}})[Z_{10} : E^{m}_{7}] \\ & - k^{+}_{z_{10}:e^{m}_{7}} e^{m}_{7} z_{10} - k^{+}_{z_9:e^{m}_{7}} e^{m}_{7} z_9 \\ & + (k^{cat}_{z_9:e^{m}_{7}} + k^{-}_{z_9:e^{m}_{7}})[Z_9 : E^{m}_{7}] \\ & + k^{cat}_{z^{m}_{7}:e_9} [Z^{m}_{7} : E_9] - e^{m}_{7} \frac{d}{dt}[PL^{s}_{a}] \frac{1}{p^{avail}_{PLAS}}\end{aligned} \quad (32)$$

$$\begin{aligned}\frac{d}{dt}[TFPI] = {} & - k^{+}_{TFPI:e_{10}} e_{10} [TFPI] + k^{-}_{TFPI:e_{10}} [TFPI : E_{10}] \\ & + k_{flow}([TFPI]^{up} - [TFPI]) \\ & - k^{+}_{TFPI:e^{hm}_{5}} e^{hm}_{5} [TFPI] \\ & + k^{-}_{TFPI:e^{hm}_{5}} [TFPI : E^{hm}_{5}] - k^{+}_{TFPI:e^{h}_{5}} e^{h}_{5} [TFPI] \\ & + k^{-}_{TFPI:e^{h}_{5}} [TFPI : E^{h}_{5}] - k^{+}_{TFPI:e^{m}_{10}} e^{m}_{10} [TFPI] \\ & + k^{-}_{TFPI:e^{m}_{10}} [TFPI : E^{m}_{10}]\end{aligned} \quad (33)$$

$$- k^{+}_{TFPI:PRO^{h}_{v10}} PRO^{h}[TFPI]$$
$$+ k^{-}_{TFPI:PRO^{h}_{v10}} [TFPI : PRO^{h}_{v10}]$$
$$- k^{+}_{TFPI:PRO^{h}_{v5}} PRO^{h}[TFPI]$$
$$+ k^{-}_{TFPI:PRO^{h}_{v5}} [TFPI : PRO^{h}_{v5}]$$

$$\frac{d}{dt}[TFPI : E_{10}] = k^{+}_{TFPI:e_{10}} e_{10}[TFPI] - k^{-}_{TFPI:e_{10}}[TFPI : E_{10}] \quad (34)$$
$$+ k^{-}_{TFPI:e_{10}:e^{m}_{7}}[TFPI : E_{10} : E^{m}_{7}]$$
$$- k^{+}_{TFPI:e_{10}:e^{m}_{7}} e^{m}_{7}[TFPI : E_{10}]$$
$$+ k_{flow}([TFPI : E_{10}]^{up} - [TFPI : E_{10}])$$
$$- k^{+}_{TFPI:e_{10}:e^{h}_{5}}[TFPI : E_{10}]e^{h}_{5}$$
$$+ k^{-}_{TFPI:e_{10}:e^{h}_{5}}[E_{10} : TFPI : E^{h}_{5}]$$
$$- k^{on}_{10}[TFPI : E_{10}]p^{avail}_{10} + k^{off}_{10}[TFPI : E^{m}_{10}]$$

$$\frac{d}{dt}[TFPI : E_{10} : E^{m}_{7}] = - k^{-}_{TFPI:e_{10}:e^{m}_{7}}[TFPI : E_{10} : E^{m}_{7}] \quad (35)$$
$$+ k^{+}_{TFPI:e_{10}:e^{m}_{7}} e^{m}_{7}[TFPI : E_{10}]$$
$$- [TFPI : E_{10} : E^{m}_{7}]\frac{d}{dt}[PL^{s}_{a}]\frac{1}{p^{avail}_{PLAS}}$$

$$\frac{d}{dt}[Z_7 : E_2] = k_{flow}([Z_7 : E_2]^{up} - [Z_7 : E_2]) + k^{+}_{z_7:e_2} e_2 z_7 \quad (36)$$
$$- (k^{cat}_{z_7:e_2} + k^{-}_{z_7:e_2})[Z_7 : E_2]$$

$$\frac{d}{dt}[Z_7 : E_{10}] = k^{+}_{z_7:e_{10}} e_{10} z_7 - (k^{cat}_{z_7:e_{10}} + k^{-}_{z_7:e_{10}})[Z_7 : E_{10}] \quad (37)$$
$$+ k_{flow}([Z_7 : E_{10}]^{up} - [Z_7 : E_{10}])$$

$$\frac{d}{dt}[Z^{m}_{7} : E_{10}] = k^{+}_{z^{m}_{7}:e_{10}} e_{10} z^{m}_{7} - (k^{cat}_{z^{m}_{7}:e_{10}} + k^{-}_{z^{m}_{7}:e_{10}})[Z^{m}_{7} : E_{10}] \quad (38)$$
$$- [Z^{m}_{7} : E_{10}]\frac{d}{dt}[PL^{s}_{a}]\frac{1}{p^{avail}_{PLAS}}$$

$$\frac{d}{dt}[Z_7^m : E_2] = k^+_{z_7^m:e_2} e_2 z_7^m - (k^{cat}_{z_7^m:e_2} + k^-_{z_7^m:e_2})[Z_7^m : E_2] - [Z_7^m : E_2]\frac{d}{dt}[PL_a^s]\frac{1}{p^{avail}_{PLAS}} \tag{39}$$

$$\begin{aligned}\frac{d}{dt}[APC] = {} & (k^{cat}_{e_5^m:APC} + k^-_{e_5^m:APC})[APC : E_5^m] - k^{cat}_{e_5^m:APC} e_5^m \\ & + (k^{cat}_{e_8^m:APC} + k^-_{e_8^m:APC})[APC : E_8^m] \\ & - k^+_{e_8^m:APC} e_8^m [APC] \\ & + k_{flow}([APC]^{up} - [APC]) \\ & - k_{diff}([APC] - [APC^{ec}]) - k^+_{e_5:APC} e_5 [APC] \\ & + (k^{cat}_{e_5:APC} + k^-_{e_5:APC})[APC{:}E_5] - k^+_{e_5^{hm}:APC} e_5^{hm} APC \\ & + (k^{cat}_{e_8:APC} + k^-_{e_8:APC})[APC : E_8] - k^+_{e_8:APC} e_8 [APC] \\ & + k^-_{e_5^{hm}:APC}[APC : E_5^{hm}] + k^{cat}_{e_5^{hm}:APC}[APC : E_5^{hm}] \\ & - k^+_{e_5^h:APC} e_5^h APC + k^-_{e_5^h:APC}[APC : E_5^h] \\ & + k^{cat}_{e_5^h:APC}[APC : E_5^h] \underline{+ \boldsymbol{k^{cat}_{pc}[TM_{RZ} : E_2 : PC]}}\end{aligned} \tag{40}$$

$$\frac{d}{dt}[Z_{10} : E_7^m] = k^+_{z_{10}:e_7^m} e_7^m z_{10} - (k^{cat}_{z_{10}:e_7^m} + k^-_{z_{10}:e_7^m})[Z_{10} : E_7^m] - [Z_{10} : E_7^m]\frac{d}{dt}[PL_a^s]\frac{1}{p^{avail}_{PLAS}} \tag{41}$$

$$\frac{d}{dt}[Z_{10}^m : TEN] = k^+_{z_{10}^m:TEN} z_{10}^m [TEN] - (k^{cat}_{z_{10}^m:TEN} + k^-_{z_{10}^m:TEN})[Z_{10^m} : TEN] \tag{42}$$

$$\frac{d}{dt}[Z_5 : E_2] = k^+_{z_5:e_2} e_2 z_5 - (k^{cat}_{z_5:e_2} + k^-_{z_5:e_2})[Z_5 : E_2] + k_{flow}([Z_5 : E_2]^{up} - [Z_5 : E_2]) \tag{43}$$

$$\frac{d}{dt}[Z_5^m : e_{10}^m] = k^+_{z_5^m:e_{10}^m} e_{10}^m z_5^m - (k^{cat}_{z_5^m:e_{10}} + k^-_{z_5^m:e_{10}^m})[Z_5^m : E_{10}^m] \tag{44}$$

$$\frac{d}{dt}[Z_5^m : E_2^m] = k^+_{z_5^m:e_2^m} e_2^m z_5^m - (k^{cat}_{z_5^m:e_2^m} + k^-_{z_5^m:e_2^m})[Z_5^m : E_2^m] \tag{45}$$

$$\frac{d}{dt}[Z_8^m : E_{10}^m] = k^+_{z_8^m:e_{10}^m} e_{10}^m z_8^m - (k^{cat}_{z_8^m:e_{10}^m} + k^-_{z_8^m:e_{10}^m})[Z_8^m : E_{10}^m] \tag{46}$$

$$\frac{d}{dt}[Z_8^m : E_2^m] = k^+_{z_8^m:e_2^m} e_2^m z_8^m - (k^{cat}_{z_8^m:e_2^m} + k^-_{z_8^m:e_2^m})[Z_8^m : E_2^m] \tag{47}$$

$$\frac{d}{dt}[Z_8 : E_2] = k^+_{z_8:e_2} e_2 z_8 - (k^{cat}_{z_8:e_2} + k^-_{z_8:e_2})[Z_8 : E_2] + k_{flow}([Z_8 : E_2]^{up} - [Z_8 : E_2]) \tag{48}$$

$$\frac{d}{dt}[APC : E_8^m] = k^+_{e_8^m:APC} e_8^m [APC] - (k^{cat}_{e_8^m:APC} + k^-_{e_8^m:APC})[APC : E_8^m] \tag{49}$$

$$\frac{d}{dt}[Z_9 : E_7^m] = k^+_{z_9:e_7^m} e_7^m z_9 - (k^{cat}_{z_9:e_7^m} + k^-_{z_9:e_7^m})[Z_9 : E_7^m] - [Z_9 : E_7^m]\frac{d}{dt}[PL_a^s]\frac{1}{p^{avail}_{PLAS}} \tag{50}$$

$$\frac{d}{dt}[Z_2^m : PRO] = k^+_{z_2^m:PRO} z_2^m [PRO] - (k^{cat}_{z_2^m:PRO} + k^-_{z_2^m:PRO})[Z_2^m : PRO] \tag{51}$$

$$\frac{d}{dt}[APC : E_5^m] = k^+_{e_5^m:APC} e_5^m [APC] - (k^{cat}_{e_5^m:APC} + k^-_{e_5^m:APC})[APC : E_5^m] \tag{52}$$

$$\frac{d}{dt}[Z_7 : E_9] = k^+_{z_7:e_9} e_9 z_7 - (k^{cat}_{z_7:e_9} + k^-_{z_7:e_9})[Z_7 : E_9] \tag{53}$$

$$\frac{d}{dt}[Z_7^m : E_9] = k^+_{z_7^m:e_9} e_9 z_7^m - (k^{cat}_{z_7^m:e_9} + k^-_{z_7^m:e_9})[Z_7^m : E_9] - [Z_7^m : E_9]\frac{d}{dt}[PL_a^s]\frac{1}{p^{avail}_{PLAS}} \tag{54}$$

$$\frac{d}{dt}[TF] = -[TF]\frac{d}{dt}[PL_a^s]\frac{1}{p^{avail}_{PLAS}} \tag{55}$$

$$\frac{d}{dt}e_9^{m*} = k_9^{on} p_9^{*,avail} e_9 - k_9^{off} e_9^{m*} + k^-_{e_8^m:e_9^m}[TEN^*] - k^+_{e_8^m:e_9^m} e_8^m e_9^{m*} - k^{AT}_{e_9^m} e_9^{m*}[AT] \tag{56}$$

$$\frac{d}{dt}[TEN^*] = -k^-_{e_8^m:e_9^m}[TEN^*] + k^+_{e_8^m:e_9^m} e_8^m e_9^{m*} + (k^{cat}_{z_{10}^m:TEN} + k^-_{z_{10}^m:TEN})[Z_{10}^m : TEN^*] + k^+_{z_{10}^m:TEN}[TEN^*]z_{10}^m \tag{57}$$

$$\frac{d}{dt}[Z_{10}^m : TEN^*] = k^+_{z_{10}^m:TEN}[TEN^*]z_{10}^m - (k^{cat}_{z_{10}^m:TEN} + k_{z_{10}^m} : TEN)^-[Z_{10}^m : TEN^*] \tag{58}$$

$$\frac{d}{dt}e_2^{ec} = k_{diff}(e_2 - e_2^{ec}) + k_{flow}(e_2^{ec,up} - e_2^{ec}) - k^{on}_{TM}e_2^{ec}[TM]^{avail} + k^{off}_{TM}[TM : E_2^{ec}] - k^{AT}_{e_2^{ec}} e_2^{ec}[AT] \tag{59}$$

$$\frac{d}{dt}[APC^{ec}] = k_{flow}([APC]^{up} - [APC^{ec}]) + k_{diff}([APC] - [APC^{ec}]) + k^{cat}_{PC:TM:e_2}[TM : E_2^{ec} : APC] \tag{60}$$

$$\frac{d}{dt}e_9^{ec} = k_{diff}(e_9 - e_9^{ec}) + k_{flow}(e_9^{up} - e_9^{ec}) - k^{AT}_{e_9^{ec}} e_9^{ec}[AT] \tag{61}$$

$$\frac{d}{dt}e_{10}^{ec} = k_{diff}(e_{10} - e_{10}^{ec}) + k_{flow}(e_{10}^{up} - e_{10}^{ec}) - k^{AT}_{e_{10}^{ec}} e_{10}^{ec}[AT] \tag{62}$$

$$\frac{d}{dt}[TM : E_2^{ec}] = k^+_{TM}[E_2^{ec}](1 - [TM : E_2^{ec}] - k^+_{PC:TM:e_2}[TM : E_2^{ec}] \tag{63}$$

$$- [TM : E_2^{ec} : APC]) - k^-_{TM}[TM : E_2^{ec}] + (k^-_{PC:TM:e_2} + k^{cat}_{PC:TM:e_2})[TM : E_2^{ec} : APC]$$

$$\frac{d}{dt}[TM : E_2^{ec} : APC] = k^+_{PC:TM:e_2}[TM : E_2^{ec}] - (k^-_{PC:TM:e_2} + k^{cat}_{PC:TM:e_2})[TM : E_2^{ec} : APC] \tag{64}$$

$$\frac{d}{dt}[APC : E_5] = -(k^{cat}_{e_5:APC} + k^-_{e_5:APC})[APC : E_5] + k^+_{e_5:APC}e_5[APC] \tag{65}$$

$$\frac{d}{dt}[APC : E_8] = -(k^{cat}_{e_8:APC} + k^-_{e_8:APC})[APC : E_8] + k^+_{e_8:APC}e_8[APC] \tag{66}$$

$$\frac{d}{dt}z_{11} = k_{flow}(z_{11}^{up} - z11) - k_{z_{11}}^{on} z_{11} p_{11}^{avail} + k_{z_{11}}^{off} z_{11}^{m}$$
$$- k^{+}_{z_{11}:e_{11}^{h}} z_{11} e_{11}^{h} + k^{-}_{z_{11}:e_{11}^{h}} [Z_{11} : E_{11}^{h}]$$
$$- k^{+}_{z_{11}:e_{11}} z_{11} e_{11} + k^{-}_{z_{11}:e_{11}} [Z_{11} : E_{11}]$$
$$- k^{+}_{z_{11}:e_{2}} z_{11} e_{2} + k^{-}_{z_{11}:e_{2}} [Z_{11} : E_{2}] \quad (67)$$

$$\frac{d}{dt}e_{11} = k_{flow}(e_{11}^{up} - e_{11}) - k_{e_{11}^{on,s}} e_{11} p_{111}^{avail} + k_{e_{11}^{off,s}} e_{11}^{m*}$$
$$- k^{+}_{z_9:e_{11}} z_9 e_{11} + (k^{-}_{z_9:e_{11}} + k^{cat}_{z_9:e_{11}})[Z_9 : E_{11}]$$
$$- k^{+}_{z_{11}:e_{11}} z_{11} e_{11} + (k^{-}_{z_{11}:e_{11}} + k^{cat}_{z_{11}:e_{11}})[Z_{11} : E_{11}]$$
$$+ k^{cat}_{e_{11}^{h}:e_{11}^{h}} [E_{11}^{h} : E_{11}^{h}] - k^{+}_{e_{11}^{h}:e_{11}} e_{11}^{h} e_{11}$$
$$+ (k^{-}_{e_{11}^{h}:e_{11}} + 2k^{cat}_{e_{11}^{h}:e_{11}})[E_{11}^{h} : E_{11}]$$
$$+ k^{cat}_{e_{11}^{h}:e_{2}} [E_{11}^{h} : E_{2}] - k^{AT}_{e_{11}} e_{11} [AT] \quad (68)$$

$$\frac{d}{dt}z_{11}^{m} = k_{z_{11}}^{on} z_{11} p_{11}^{avail} - k_{z_{11}}^{off} z_{11}^{m} - k^{+}_{z_{11}^{m}:e_{11}^{h,m}} z_{11}^{m} e_{11}^{h,m}$$
$$+ k^{-}_{z_{11}^{m}:e_{11}^{h,m}} [Z_{11}^{m} : E_{11^{hm}}] - k^{+}_{z_{11}^{m}:e_{11}^{m*}} z_{11}^{m} e_{11}^{m*}$$
$$+ k^{-}_{z_{11}^{m}:e_{11}^{m*}} [Z_{11}^{m} : E_{11}^{m*}] - k^{+}_{z_{11}^{m}:E_{2}^{m}} z_{11}^{m} e_{2}^{m}$$
$$+ k^{-}_{z_{11}^{m}:e_{2}^{m}} [Z_{11}^{m} : E_{2}^{m}] \quad (69)$$

$$\frac{d}{dt}e_{11}^{m*} = k_{e_{11}}^{on*} e_{11} p_{111}^{avail} - k_{e_{11}}^{off*} e_{11}^{m*} - k^{+}_{z_9^{m}:e_{11}^{m*}} e_{11}^{m*} z_9^{m}$$
$$+ (k^{-}_{z_9^{m}:e_{11}^{m*}} + k^{cat}_{z_9^{m}:e_{11}^{m*}})[Z_9^{m} : E_{11}^{m*}]$$
$$- k^{+}_{z_{11}^{m}:e_{11}^{m*}} z_{11}^{m} e_{11}^{m*} + (k^{-}_{z_{11}^{m}:e_{11}^{m*}}$$
$$+ k^{cat}_{z_{11}^{m}:e_{11}^{m*}})[Z_{11}^{m} : E_{11}^{m*}] + k^{+}_{e_{11}^{h,m*}:e_{11}^{m*}} e_{11}^{h,m*} e_{11}^{m*}$$
$$+ (k^{-}_{e_{11}^{h,m*}:e_{11}^{m*}} + 2k^{cat}_{e_{11}^{h,m*}:e_{11}^{m*}})[E_{11}^{hms} : E_{11}^{h,m}]$$
$$+ k^{cat}_{e_{11}^{h,m*}:e_{2}^{m}} [E_{11}^{hms} : E_{2}^{m}] + k^{AT}_{e_{11}} e_{11}^{m*} [AT] \quad (70)$$

$$\frac{d}{dt}[Z_{11}^{m} : E_{2}^{m}] = k^{+}_{z_{11}^{m}:e_{2}^{m}} z_{11}^{m} e_{2}^{m} - (k^{-}_{z_{11}^{m}:e_{2}^{m}} + k^{cat}_{z_{11}^{m}:e_{2}^{m}})[Z_{11}^{m} : E_{2}^{m}] \quad (71)$$

$$\frac{d}{dt}[Z_9^{m} : E_{11}^{m*}] = k^{+}_{z_9^{m}:e_{11}^{m*}} z_9^{m} e_{11}^{m} s - (k^{-}_{z_9^{m}:e_{11}^{m*}} + k^{cat}_{z_9^{m}:e_{11}^{m*}})[Z_9^{m} : E_{11}^{m*}] \quad (72)$$

$$\frac{d}{dt}[Z_{11}:E_2] = k_{flow}([Z_{11}:E_2]^{up} - [Z_{11}:E_2]) + k^{+}_{z_{11}:e_2} z_{11} e_2 \\ - (k^{-}_{z_{11}:e_2} + k^{cat}_{z_{11}:e_2})[Z_{11}:E_2] \tag{73}$$

$$\frac{d}{dt}[Z_9:E_{11}] = k_{flow}([Z_9:E_{11}]^{up} - [Z_9:E_{11}]) + k^{+}_{z_9:e_{11}} z_9 e_{11} \\ - (k^{-}_{z_9:E_{11}} + k^{cat}_{z_9:e_{11}})[Z_9:E_{11}] \tag{74}$$

$$\frac{d}{dt}[Z_{11}:E_{11}] = k_{flow}([Z_{11}:E_{11}]^{up} - [Z_{11}:E_{11}]) \\ + k^{+}_{z_{11}:e_{11}} z_{11} e_{11} - (k^{-}_{z_{11}:e_{11}} + k^{cat}_{z_{11}:e_{11}})[Z_{11}:E_{11}] \tag{75}$$

$$\frac{d}{dt}[Z_9:E^h_{11}] = k_{flow}([Z_9:E^h_{11}]^{up} - [Z_9:E^h_{11}]) + k^{+}_{z_9:e^h_{11}} z_9 e^h_{11} \\ - (k^{-}_{z_9:e^h_{11}} + k^{cat}_{z_9:e^h_{11}})[Z_9:E^h_{11}] \tag{76}$$

$$\frac{d}{dt}[Z^m_9:E^{h,m}_{11}] = k^{+}_{z^m_9:e^{h,m}_{11}} z^m_9 e^{h,m}_{11} - (k^{-}_{z^m_9:e^h_{11}m} + k^{cat}_{z^m_9:e^h_{11}m})[Z^m_9:E^{h,m}_{11}] \tag{77}$$

$$\frac{d}{dt}[Z_{11}:E^h_{11}] = k_{flow}([Z_{11}:E^h_{11}]^{up} - [Z_{11}:E^h_{11}]) \\ + k_{z_{11}:e^{hp}_{11}} z_{11} e^h_{11} - (k^{-}_{z_{11}:e^h_{11}} + k^{cat}_{z_{11}:e^h_{11}})[Z_{11}:E^h_{11}] \tag{78}$$

$$\frac{d}{dt}[E^h_{11}:E^h_{11}] = k_{flow}([E^h_{11}:E^h_{11}]^{up} - [E^h_{11}:E^h_{11}]) \\ + k^{+}_{e^h_{11}:e^h_{11}} e^h_{11} e^h_{11} - (k^{-}_{e^h_{11}:e^h_{11}} + k^{cat}_{e^h_{11}:e^h_{11}})[E^h_{11}:E^h_{11}] \tag{79}$$

$$\frac{d}{dt}[E^h_{11}:E_{11}] = k_{flow}([E^h_{11}:E_{11}]^{up} - [E^h_{11}:E_{11}]) + k^{+}_{e^h_{11}:e_{11}} e^h_{11} \\ - (k^{-}_{e^h_{11}:e_{11}} + k^{cat}_{e^h_{11}:e_{11}})[E^h_{11}:E_{11}] \tag{80}$$

$$\frac{d}{dt}[E^h_{11}:E_2] = k_{flow}([E^h_{11}:E_2]^{up} - [E^h_{11}:E_2]) + k^{+}_{e^h_{11}:e_2} e^h_{11} e_2 \\ - (k^{-}_{e^h_{11}:e_2} + k^{cat}_{e^h_{11}:e_2})[E^h_{11}:E_2] \tag{81}$$

$$\frac{d}{dt}[Z^m_{11}:E^{h,m}_{11}] = k^{+}_{z^m_{11}:e^{h,m}_{11}} z^m_{11} e^{h,m}_{11} - (k^{-}_{z^m_{11}:e^{h,m}_{11}} + k^{cat}_{z^m_{11}:e^{h,m}_{11}})[Z^m_{11}:E^{h,m}_{11}] \tag{82}$$

$$\frac{d}{dt}[Z^m_{11}:E^{m*}_{11}] = k^{+}_{z^m_{11}:e^{m*}_{11}} z^m_{11} e^{m*}_{11} - (k^{-}_{z^m_{11}:e^{m*}_{11}} + k^{cat}_{z^m_{11}:e^m_{11}s})[Z^m_{11}:E^{m*}_{11}] \tag{83}$$

$$\frac{d}{dt}[E_{11}^{hms} : E_{11}^{h,m}] = k^{+}_{e_{11}^{h,m*}:e_{11}^{h,m}} e_{11}^{h,m} - (k^{-}_{e_{11}^{h,m*}:e_{11}^{h,m}} + k^{cat}_{e_{11}^{h,m*}:e_{11}^{h,m}})[E_{11}^{hms} : E_{11}^{h,m}] \tag{84}$$

$$\frac{d}{dt}[E_{11}^{hms} : E_{11}^{m*}] = k_{e_{11}^{h,m*}:e_{11}^{h,m*}} e_{11}^{m*} - (k^{-}_{e_{11}^{h,m*}:e_{11}^{m*}} + k^{cat}_{e_{11}^{h,m*}:e_{11}^{m*}})[E_{11}^{hms} : E_{11}^{m*}] \tag{85}$$

$$\frac{d}{dt}[E_{11}^{hms} : E_{2}^{m}] = k^{+}_{e_{11}^{h,m*}:e_{2}^{m}} e_{11}^{h,m*} e_{2}^{m} - (k^{-}_{e_{11}^{hms:e_2^m}} + k^{cat}_{e_{11}^{h,m*}:e_{2}^{m}}[E_{11}^{hms} : E_{2}^{m}]) \tag{86}$$

$$\begin{aligned}\frac{d}{dt}e_{11}^{h} = {} & k^{on*}_{e_{11}^{h}} e_{11}^{h} p_{111}^{avail} + k^{off*}_{e_{11}^{h}} e_{11}^{h,m*} - k^{on}_{e_{11}^{h}} e_{11}^{h} p_{11}^{avail} \\ & + k^{off}_{e_{11}^{h}} e_{11}^{h,m} - k^{+}_{z_9:e_{11}^{h}} z_9 e_{11}^{h} + (k^{-}_{z_9:e_{11}^{h,m}} + k^{cat}_{z_9:e_{11}^{h}})[Z_9 : E_{11}^{h}] \\ & - k_{z_{11}:e_{11}^{hp}} z_{11} e_{11}^{h} + (k^{-}_{z_{11}:e_{11}^{h}m} + 2k^{cat}_{z_{11}:e_{11}^{h}})[Z_{11:E_{11}^{h}}] \\ & + k^{cat}_{z_{11}:e_{11}}[Z_{11} : E_{11}] + k^{cat}_{z_{11}:e_{2}}[Z_{11} : E_{2}] \\ & - 2k^{+}_{e_{11}^{h}:e_{11}^{h}} e_{11}^{h} e_{11}^{h} + (2k^{-}_{e_{11}^{h}:e_{11}^{h}} + k^{cat}_{e_{11}^{g}:e_{11}^{h}})[E_{11}^{h} : E_{11}^{h}] \\ & - k^{+}_{e_{11}^{h}:e_{11}} e_{11}^{h} e_{11} + k^{-}_{e_{11}^{h}:e_{11}}[E_{11}^{h} : E_{11}] \\ & - k^{+}_{e_{11}^{h}:e_{2}} e_{11}^{h} e_{2} + k^{-}_{e_{11}^{h}:e_{2}}[E_{11}^{h} : E_{2}] \\ & + k_{flow}(e_{11}^{h,up} - e_{11}^{h}) - k^{AT}_{e_{11}^{h}} e_{11}^{h}[AT]\end{aligned} \tag{87}$$

$$\begin{aligned}\frac{d}{dt}e_{11}^{h,m} = {} & k^{on}_{e_{11}^{h}} e_{11}^{h} p_{11}^{avail} - k^{off}_{e_{11}^{h}} e_{11}^{h,m} - k^{+}_{z_9^m:e_{11}^{h,m}} z_9^m e_{11}^{h,m} \\ & + (k^{-}_{z_9^m:e_{11}^{h,m}} + k^{cat}_{z_9^m:e_{11}^{h,m}})[Z_9^m : E_{11}^{h,m}] \\ & + (k^{-}_{z_{11}^m:e_{11}^{h,m}} + 2k^{cat}_{z_{11}^m:e_{11}^{h,m}})[Z_{11}^m : E_{11}^{h,m}] \\ & - k^{+}_{z_{11}^m:e_{11}^{h,m}} z_{11}^m e_{11}^{h,m} + k^{cat}_{z_{11}^m:e_{11}^{m*}}[Z_{11}^m : E_{11}^{m*}] \\ & + k^{cat}_{z_{11}^m:e_2^m}[Z_{11}^m : E_2^m] - k^{+}_{e_{11}^{h,m*}:e_{11}^{h,m}} e_{11}^{h,m*} e_{11}^{h,m} \\ & + (k^{-}_{e_{11}^{h,m*}:e_{11}^{h,m}} + k^{cat}_{e_{11}^{h,m*}:e_{11}^{h,m}})[E_{11}^{hms} : E_{11}^{h,m}] - k^{AT}_{e_{11}} e_{11}^{hm}[AT]\end{aligned} \tag{88}$$

$$\begin{aligned}\frac{d}{dt}e_{11}^{h,m*} =& k_{e_{11}^h}^{on*} e_{11}^h p_{111}^{avail} - k_{e_{11}^h}^{off*} e_{11}^{h,m*} \\ &- k_{e_{11}^{h,m*}:e_{11}^{h,m}}^{+} e_{11}^{h,m*} e_{11}^{h,m} + k_{e_{11}^{h,m*}:e_{11}^{h,m}}^{-} [E_{11}^{hms} : E_{11}^{h,m}] \\ &- k_{e_{11}^{h,m*}:e_{11}^{m*}}^{+} e_{11}^{h,m*} e_{11}^{m*} + k_{e_{11}^{h,m*}:e_{11}^{m}s}^{-} [E_{11}^{hms} : E_{11}^{m*}] \\ &- k_{e_{11}^{h,m*}:e_2^m}^{+} e_{11}^{h,m*} e_2^m + k_{e_{11}^{h,m*}:e_2^m}^{-} [E_{11}^{hms} : E_2^m]\end{aligned} \tag{89}$$

$$\begin{aligned}\frac{d}{dt}e_5^{hm} =& k_{z_5^m:e_{10}^m}^{cat} [Z_5^m : E_{10}^m] + k_5^{on} e_5^h p_5^{avail} - k_5^{off} e_5^{hm} \\ &- k_{e_5^{hm}:e_{10}^m}^{+} e_{10}^m e_5^{hm} + k_{e_5^{hm}:e_{10}^m}^{-} PRO^h \\ &- k_{e_5^{hm}:e_2^m}^{+} e_2^m e_5^{hm} + k_{e_5^{hm}:e_2^m}^{-} [E_5^{hm} : E_2^m] \\ &- k_{TFPI:e_5^{hm}}^{+} e_5^{hm} TFPI + k_{TFPI:e_5^{hm}}^{-} [TFPI : E_5^{hm}] \\ &- k_{e_5^{hm}:APC}^{+} e_5^{hm} APC + k_{e_5^{hm}:APC}^{-} [APC : E_5^{hm}] \\ &- k_{TFPI:e_{10}:e_5^{hm}}^{+} [TFPI : E_{10}^m] e_h^{hm} \\ &+ k_{TFPI:e_{10}:e_5^{hm}}^{-} [E_{10}^m : TFPI : E_5^{hm}] \\ &- k_{TFPI:e_{10}:e_5^{hm}:e_{10}^m}^{+} [TFPI : E_{10}^m] e_5^{hm} \\ &+ k_{TFPI:e_{10}:e_5^{hm}:e_{10}^m}^{-} [TFPI : PRO_{v10}^h]\end{aligned} \tag{90}$$

$$\begin{aligned}\frac{d}{dt}e_5^h =& - k_{5on} e_5^h p_5^{avail} + k_5^{off} e_5^{hm} + k_{flow}(e_5^{up} - e_5^h) \\ &+ (1 - f_5) N_5 dpl \cdot p - k_{e_5^h:e_2}^{+} e_5^h \\ &+ k_{e_5^h:e_2}^{-} [E_5^h : E_2] - k_{e_5^h:APC}^{+} APC \cdot e_5^h \\ &+ k_{e_5^h:APC}^{-} [APC : E_5^h] - k_{TFPI:e_5^h}^{+} e_5^h TFPI \\ &+ k_{TFPI:e_5^h}^{-} [TFPI\colon E_5^h] - k_{TFPI:e_{10}:e_5^h}^{+} [TFPI\colon E_{10}] e_5^h \\ &+ k_{TFPI:e_{10}:e_5^h}^{-} [E_{10} : TFPI : E_5^h] \\ &- k_{TFPI:e_{10}^m:e_5^h}^{+} [TFPI : E_{10}^m] e_5^h \\ &+ k_{TFPI:e_{10}^m:e_5^h}^{-} [E_{10}^m : TFPI : E_5^h]\end{aligned} \tag{91}$$

$$\frac{d}{dt}PRO^h = k^+_{e_5^{hm}:e_{10}^m} e_{10}^m e_5^{hm} - k^-_{e_5^{hm}:e_{10}^m} PRO^h \tag{92}$$
$$- k^+_{z_2^m:PRO^h} PRO^h z_2^m + k^-_{z_2^m:PRO^h} [Z_2^m : PRO^h]$$
$$+ k^{cat}_{z_2^m:PRO^h} [Z_2^m : PRO^h]$$
$$- k^+_{TFPI:PRO^h_{v10}} PRO^h [TFPI]$$
$$+ k^-_{TFPI:PRO^h_{v10}} [TFPI : PRO^h_{v10}]$$
$$- k^+_{TFPI:PRO^h_{v5}} PRO^h [TFPI] k^-_{TFPI:PRO^h_{v5}}$$
$$[TFPI : PRO^h_{v5}]$$
$$- k^+_{PRO^h:e_2^m} PRO^h e_2^m + k^-_{PRO^h:e_2^m} [PRO^h : E_2^m]$$

$$\frac{d}{dt}[Z_2^m : PRO^h] = k^+_{z_2^m:PRO^h} PRO^h z_2^m - k^-_{z_2^m:PRO^h} [Z_2^m : PRO^h] \tag{93}$$
$$- k^{cat}_{z_2^m:PRO^h} [Z_2^m PRO^h]$$

$$\frac{d}{dt}[E_5^{hm} : E_2^m] = k^+_{e_5^{hm}:e_2^m} e_2^m e_5^{hm} - k^-_{e_5^{hm}:e_2^m} [E_5^{hm} : E_2^m] \tag{94}$$
$$- k^{cat}_{e_5^{hm}:e_2^m} [E_5^{hm} : E_2^m]$$

$$\frac{d}{dt}[E_5^h : E_2] = + k^+_{e_5^h:e_2} e_2 e_5^h - k^-_{TFPI:e_5^h} [E_5^h : E_2] \tag{95}$$
$$- k^{cat}_{e_5^h:e_2} [E_5^h : E_2] + k_{flow}([E_5^h : E_2]^{up} - [E_5^h : E_2])$$

$$\frac{d}{dt}[TFPI : E_5^{hm}] = k^+_{TFPI:e_5^{hm}} e_5^{hm} TFPI \tag{96}$$
$$- k^-_{TFPI:e_5^{hm}} [TFPI : E_5^{hm}]$$
$$- k^+_{TFPI:e_5^{hm}:e_{10}m} [TFPI : E_5^{hm}] e_{10}^m$$
$$+ k^-_{TFPI:e_5^{hm}:e_{10}m} [E_{10}^m : TFPI : E_5^{hm}]$$
$$+ k_5^{on} [TFPI : E_5^h] p_5^{avail} - k_5^{off} [TFPI : E_5^{hm}]$$
$$- k^+_{TFPI:e_{10}:e_5^{hm}} [TFPI : E_5^{hm}] e_{10}^m$$
$$+ k^-_{TFPI:e_{10}:e_5^{hm}} [TFPI : PRO^h_{v5}]$$

$$- k^{+}_{TFPI:e_5^{hm}:e_{10}}[TFPI : E_5^{hm}]e_{10}$$

$$+ k^{-}_{TFPI:e_5^{hm}:e_{10}}[E_{10} : TFPI : E_5^{hm}]$$

$$\frac{d}{dt}[APC : E_5^{hm}] = k^{+}_{e_5^{hm}:APC} E_5^{hm} APC - k^{-}_{e_5^{hm}:APC}[APC : E_5^{hm}] \quad (97)$$

$$+ k^{cat}_{e_5^{hm}:APC}[APC : E_5 hm]$$

$$\frac{d}{dt}[APC : E_5^{h}] = k^{+}_{e_5^{h}:APC} e_5^{h} APC - k^{-}_{e_5^{hm}:APC}[APC : E_5^{h}] \quad (98)$$

$$- k^{cat}_{e_5^{h}:APC}[APC : E_5^{h}]$$

$$+ k_{flow}([APC : E_5^{h}]^{up} - [APC : E_5^{h}])$$

$$\frac{d}{dt}[TFPI : E_5^{h}] = k_{TFPI:e_5^{h}up} e_5^{h} TFPI - k^{-}_{kTFPI:e_5^{h}}[TFPI : E_5^{h}] \quad (99)$$

$$+ k_{flow}([TFPI : E_5^{h}]^{up} - [TFPI : E_5^{h}])$$

$$- k^{+}_{TFPI:e_5^{h}:e_{10}}[TFPI : E_5^{h}]e_{10}$$

$$+ k^{-}_{TFPI:e_5^{h}:e_{10}}[E_{10} : TFPI : E_5^{h}]$$

$$+ k_5^{on}[TFPI : E_5^{h}]p_5^{avail} + k_5^{off}[TFPI : E_5^{hm}]$$

$$\frac{d}{dt}[TFPI : E_{10}^{m}] = k^{+}_{TFPI:e_{10}^{m}} e_{10}^{m} TFPI - k^{-}_{TFPI:e_{10}^{m}}[TFPI : E_{10}^{m}] \quad (100)$$

$$- k^{+}_{TFPI:e_{10}:e_5^{h}m}[TFPI : E_{10}^{m}]e_5^{hm}$$

$$+ k^{-}_{TFPI:e_{10}:e_5^{hm}}[E_{10}^{m} : TFPI : E_5^{hm}]$$

$$+ k_{10}^{on}[TFPI : E_{10}]p_{10}^{avail} - k_{10}^{off}[TFPI : E_{10}^{m}]$$

$$- k^{+}_{TFPI:e_{10}^{m}:e_5^{hm}}[TFPI : E_{10}^{m}]e_5^{hm}$$

$$+ k^{-}_{TFPI:e_{10}^{m}:e_5^{hm}}[TFPI : PRO_{v10}^{h}]$$

$$- k^{+}_{TFPI:e_{10}^{m}:e_5^{h}}[TFPI : E_{10}^{m}]e_5^{h}$$

$$+ k^{-}_{TFPI:e_{10}^{m}:e_5^{h}}[E_{10}^{m} : TFPI : E_5^{h}]$$

$$\frac{d}{dt}[TFPI : PRO^h_{v10}] = k^+_{TFPI:PRO^h_{v10}} PRO^h[TFPI] \tag{101}$$
$$- k^-_{TFPI:PRO^h_{v10}}[TFPI : PRO^h_{v10}]$$
$$+ k^+_{TFPI:e^m_{10}:e^{hm}_5}[TFPI : E^p_{10}][TFPI : E^m_{10}]e^{hm}_5$$
$$- k^-_{TFPI:e^m_{10}:e^{hm}_5}[TFPI : PRO^h_{v10}]$$

$$\frac{d}{dt}[TFPI : PRO^h_{v5}] = k^+_{TFPI:PRO^h_{v5}} PRO^h[TFPI] \tag{102}$$
$$- k^-_{TFPI:PRO^h_{v5}}[TFPI : PRO^h_{v5}]$$
$$+ k^+_{TFPI:e_{10}:e^{hm}_5}[TFPI : E^{hm}_5]e^m_{10}$$
$$- k^-_{TFPI:e_{10}:e^{hm}_5}[TFPI : PRO^h_{v5}]$$

$$\frac{d}{dt}[E^m_{10} : TFPI : E^{hm}_5] = k^+_{TFPI:e_{10}:e^h_5m}[TFPI : E^m_{10}]e^{hm}_5 \tag{103}$$
$$- k^-_{TFPI:e_{10}:e^h_5m}[E^m_{10} : TFPI : E^{hm}_5]$$
$$+ k^+_{TFPI:e^{hm}_5:e_{10}m}[TFPI : E^{hm}_5]e^m_{10}$$
$$- k^-_{TFPI:e^{hm}_5:e_{10}m}[E^m_{10} : TFPI : E^{hm}_5]$$
$$+ k^{ont}_{10} p^{avail}_{10} - k^{offt}_{10}[E^m_{10} : TFPI : E^{hm}_5]$$
$$+ k^{ont}_5[E^m_{10} : TFPI : E^h_5]p^{avail}_5$$
$$- k^{offt}_5[E^m_{10} : TFPI : E^{hm}_5]$$

$$\frac{d}{dt}[E_{10} : TFPI : E^h_5] = k^+_{TFPI:e_{10}:e^h_5}[TFPI : E_{10}]e^h_5 \tag{104}$$
$$- k^-_{TFPI:e_{10}:e^h_5}[E_{10} : TFPI : E^h_5]$$
$$+ k^+_{TFPI:e^h_5:e_{10}}[TFPI : E^h_5]e_{10}$$
$$- k^-_{TFPI:e^h_5:e_{10}}[E_{10} : TFPI : E^h_5]$$
$$- k^{ont}_5[E_{10} : TFPI : E^h_5]p^{avail}_5$$
$$+ k^{offt}_5[E_{10} : TFPI : E^{hm}_5]$$
$$- k^{ont}_{10}[E_{10} : TFPI : E^h_5]p^{avail}_{10}$$

$$+ k_{10}^{offt}[E_{10}^{m} : TFPI : E_5^h]$$
$$+ k_{flow}([E_{10} : TFPI : E_5^h]^{up} - [E_{10} : TFPI : E_5^h])$$

$$\frac{d}{dt}[E_{10} : TFPI : E_5^{hm}] = k_5^{ont}[E_{10} : TFPI : E_5^h]p_5^{avail} \tag{105}$$
$$- k_5^{offt}[E_{10} : TFPI : E_5^{hm}]$$
$$- k_{10}^{ont}[E_{10} : TFPI : E_5^{hm}]p_{10}^{avail}$$
$$+ k_{10}^{offt}[E_{10}^m : TFPI : E_5^{hm}]$$
$$+ k^{+}_{TFPI:e_5^{hm}:e_{10}}[TFPI : E_5^{hm}]e_{10}$$
$$- k^{-}_{TFPI:e_5^{hm}:e_{10}}[E_{10} : TFPI : E_5^{hm}]$$

$$\frac{d}{dt}[E_{10}^m : TFPI : E_5^h] = k_{10}^{ont}[E_{10} : TFPI : E_5^h]p_{10}^{avail} \tag{106}$$
$$- k_{10}^{offt}[E_{10}^m : TFPI : E_5^h]$$
$$- k_5^{ont}[E_{10}^m : TFPI : E_5^h]p_5^{avail}$$
$$+ k_5^{offt}[E_{10}^m : TFPI : E_5^{hm}]$$
$$+ k^{+}_{TFPI:e_{10}^m:e_5^h}[TFPI : E_{10}^m]e_5^h$$
$$- k^{-}_{TFPI:e_{10}^m:e_5^h}[E_{10}^m : TFPI : E_5^h]$$

$$\frac{d}{dt}[PRO^h : E_2^m] = k^{+}_{PRO^h:e_2^m} PRO^h E_2^m \tag{107}$$
$$- k^{-}_{PRO^h:e_2^m}[PRO^h : E_2^m] - k^{cat}_{PRO^h:e_2^m}[PRO^h : E_2^m]$$

$$\frac{d}{dt}[E_9 : AT] = - k_9^{on} p_9^{avail}[E_9 : AT] + k_9^{off}[E_9^m : AT] \tag{108}$$
$$+ k_{e_9}^{AT} e_9[AT] + k_{flow}([E_9 : AT]_{up} - [E_9 : AT])$$
$$- k_9^{on} p_{91}^{avail}[E_9 : AT] + k_9^{off}[E_9^{m*} : AT]$$

$$\frac{d}{dt}[E_9^m : AT] = k_{e_9^m}^{AT} e_9^m[AT] - k_9^{off}[E_9^m : AT] \tag{109}$$
$$+ k_9^{on} p_9^{avail}[E_9 : AT]$$

$$\frac{d}{dt}[E_9^{m*} : AT] = k_{e_9^m}^{AT} e_9^{m*}[AT] - k_9^{off}[E_9^{m*} : AT] \tag{110}$$
$$+ k_9^{on} p_{91}^{avail}[E_9 : AT]$$

$$\frac{d}{dt}[E_{10} : AT] = k_{e_{10}}^{AT} e_{10}[AT] + k_{flow}([E_{10} : AT]_{up} - [E_{10} : AT]) \tag{111}$$
$$+k_{10}^{off}[E_{10}^{m} : AT] - k_{10}^{on} p_{10}^{avail}[E_{10} : AT]$$

$$\frac{d}{dt}[E_{10}^{m} : AT] = +\, k_{e_{10}^{m}}^{AT} e_{10}^{m}[AT] - k_{10}^{off}[E_{10}^{m} : AT] \tag{112}$$
$$+\, k_{10}^{on} p_{10}^{avail}[E_{10} : AT]$$

$$\frac{d}{dt}[E_2 : AT] = +\, k_{e_2}^{off}[E_2^{m} : AT] - k_{e_2}^{on} p_2^{avail}[E_2 : AT] \tag{113}$$
$$+\, k_{e_2}^{AT} e_2 + k_{flow}([E_2 : AT]_{up} - [E_2 : AT])$$

$$\frac{d}{dt}[E_2^{m} : AT] = k_{e_2^{m}}^{AT} e_2^{m}[AT] - k_{e_2}^{off}[E_2^{m} : AT] \tag{114}$$
$$+\, k_{e_2}^{on} p_2^{avail}[E_2 : AT]$$

$$\frac{d}{dt}[E_{11} : AT] = k_{e_{11}}^{AT} e_{11}[AT] - k_{e_{11}}^{AT}[E_{11} : AT][AT] \tag{115}$$
$$+\, k_{11}^{off}[E_{11}^{m*} : AT] - k_{11}^{on} p_{111}^{avail}[E_{11} : AT]$$

$$\frac{d}{dt}[AT : E_{11} : AT] = k_{e_{11}}^{AT}[E_{11} : AT][AT] \tag{116}$$

$$\frac{d}{dt}[E_{11}^{m*} : AT] = k_{e_{11}}^{AT} e_{11}^{m*}[AT] - k_{11}^{off}[E_{11}^{m*} : AT] \tag{117}$$
$$+\, k_{11}^{on} p_{111}^{avail}[E_{11} : AT]$$

$$\frac{d}{dt}[E_{11}^{h} : AT] = k_{e_{11}}^{AT} e_{11}^{h}[AT] + k_{11}^{off}[E_{11}^{hm} : AT] \tag{118}$$
$$-\, k_{11}^{on} p_{11}^{avail}[E_{11}^{h} : AT]$$

$$\frac{d}{dt}[E_{11}^{hm} : AT] = k_{e_{11}}^{AT} e_{11}^{hm}[AT] - k_{11}^{off}[E_{11}^{hm} : AT] \tag{119}$$
$$+\, k_{11}^{on} p_{11}^{avail}[E_{11}^{h} : AT]$$

$$\frac{d}{dt}[AT] = -\, k_{e_9}^{AT} e_9[AT] - k_{e_9^{m}}^{AT} e_9^{m}[AT] + k_{e_9^{m}}^{AT} e_9^{m*}[AT] \tag{120}$$
$$-\, k_{e_{10}}^{AT} e_{10}[AT] + ke_{10}^{m\,AT} e_{10}^{m}[AT] - k_{e_2}^{AT} e_2[AT]$$
$$-\, k_{e_2^{m}}^{AT} e_2^{m}[AT] - k_{e_{11}}^{AT} e_{11}[AT] - k_{e_{11}}^{AT}[E_{11} : AT][AT]$$
$$-\, k_{e_{11}}^{AT} e_{11}^{m*}[AT] - k_{e_{11}}^{AT} e_{11}^{h}[AT] - k_{e_{11}}^{AT} e_{11}^{h}[AT]$$
$$+\, k_{flow}([AT]_{up} - [AT])$$

$$\underline{\frac{d}{dt}[TM_{RZ} : E_2]} = \underline{k_{TM_{RZ}}^{\text{on}} e_2 TM_{RZ}^{avail} - k_{TM_{RZ}}^{\text{off}}[TM_{RZ} : E_2]} \tag{121}$$
$$\underline{- k_{pc}^{+}[TM_{RZ} : E_2][PC]}$$
$$\underline{+ (k_{pc}^{-} + k_{pc}^{cat})[TM_{RZ} : E_2 : PC]}$$

$$\underline{\frac{d}{dt}[TM_{RZ} : E_2 : PC]} = \underline{k_{pc}^{+}[TM_{RZ} : E_2][PC]}$$
$$\underline{- (k_{pc}^{-} + k_{pc}^{cat})[TM_{RZ} : E_2 : PC]} \tag{122}$$
